# Angucycline Glycosides from an Intertidal Sediments Strain *Streptomyces* sp. and Their Cytotoxic Activity against Hepatoma Carcinoma Cells

**DOI:** 10.3390/md16120470

**Published:** 2018-11-27

**Authors:** Aihong Peng, Xinying Qu, Fangyuan Liu, Xia Li, Erwei Li, Weidong Xie

**Affiliations:** 1 Department of Pharmacy, College of Marine Science, Shandong University, Weihai 264209, China; pengahsdu@163.com (A.P.); quxinying321@163.com (X.Q.); fangyuan617@outlook.com (F.L.); xiali@sdu.edu.cn (X.L.); 2 State Key Laboratory of Mycology, Institute of Microbiology, Chinese Academy of Sciences, Beijing 100101, China

**Keywords:** *Streptomyces*, angucycline glycosides, saquayamycin, cytotoxicity, apoptosis, SMMC-7721

## Abstract

Four angucycline glycosides including three new compounds landomycin N (**1**), galtamycin C (**2**) and vineomycin D (**3**), and a known homologue saquayamycin B (**4**), along with two alkaloids 1-acetyl-β-carboline (**5**) and indole-3-acetic acid (**6**), were isolated from the fermentation broth of an intertidal sediments-derived *Streptomyces* sp. Their structures were established by IR, HR-ESI-MS, 1D and 2D NMR techniques. Among the isolated angucyclines, saquayamycin B (**4**) displayed potent cytotoxic activity against hepatoma carcinoma cells HepG-2, SMMC-7721 and plc-prf-5, with IC_50_ values 0.135, 0.033 and 0.244 μM respectively, superior to doxorubicin. Saquayamycin B (**4**) also induced apoptosis in SMMC-7721 cells as detected by its morphological characteristics in 4′,6-diamidino-2-phenylindole (DAPI) staining experiment.

## 1. Introduction

Angucycline is a group of aromatic polyketides containing a benz[a]anthraquinone framework of the aglycone which is mostly attached with C-glycosidic moiety [1]. Naturally occurring angucyclines are exclusively produced by terrestrial and marine actinomycetes, especially *Streptomycetes* species, in which a decaketide initially derived from acetyl-CoA is catalytically cyclized to four-ring core of angucycline by polyketide cyclase [2]. The structures of angucycline glycosides always vary in the oxidation degree of aglycones along with the number and position of diverse deoxy sugars [1,2,3,4]. In some cases, e.g., galtamycin B [5], grincamycin B [6], and vineomycin B_2_ [7], the angular four-ring of typical angucycline is rearranged to linear tetracyclic or tricyclic system by enzymatic or non-enzymatic modification. Although firstly discovered half a century ago and possessing potent antibacterial, antiproliferative, and cytotoxic activities [6,7,8,9,10,11], so far, none of angucycline compounds has been successfully developed into clinical drug due to toxicity or solubility issues, which is unlike their biogenetic relatives tetracycline and anthracycline antibiotics [2]. Recent researches on angucyclines mainly concentrated on the understanding of their biosynthetic pathways in order to obtain modified analogues with medicinal potentiality through genetic manipulation [12,13,14].

**Figure 1:**
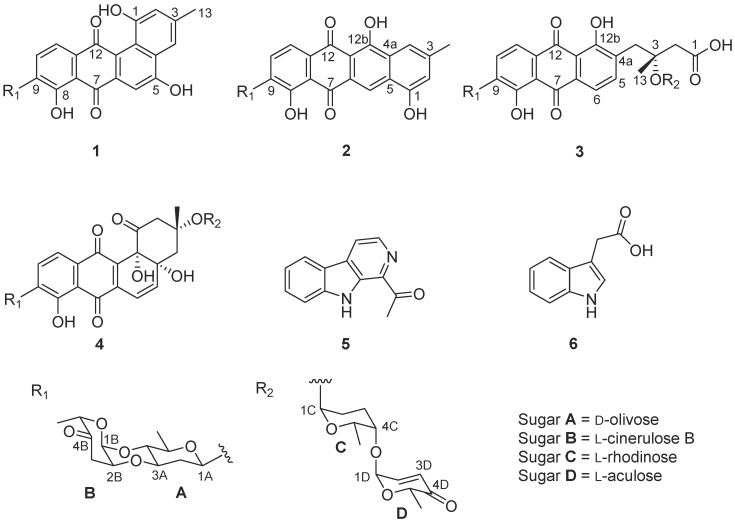
Structures of **1**–**6**.

Intertidal ecosystems are significantly different from those of seafloor. Regular tide immersion and emersion result in the dissolution of more organic carbon as well as oxygen and sulfate into intertidal sediment, which is beneficial to microbes’ survival, particularly to aerobic actinomycetes. Both metagenomes and culture-dependent isolation have verified the abundance and diversity of Actinobacteria in intertidal sediment [15]. Thus, we exploited the Actinobacteria resources from the intertidal sediment of Xiaoshi Island in Weihai, China, to screen for new antitumor agents. As a result, a *Streptomyces* sp., designated OC1610.4, was obtained, and its 16S rRNA nucleotide sequence (Accession no. MK045847) shared only 81.8% and 81.6% similarity, respectively, with those of *Streptomyces chromofuscus* (FJ486284) and *Streptomyces lannensis* (KM370050) in GenBank. The thin layer chromatography (TLC) analysis of its EtOAc extract of liquid culture medium displayed several yellow and brown spots, presumably due to aromatic polyketides. Subsequent large-scale fermentation and chromatographic isolation led to the identification of four angucycline glycosides including three new compounds, namely landomycin N (**1**), galtamycin C (**2**) and vineomycin D (**3**), and the previously reported saquayamycin B (**4**) (Figure 1), along with two alkaloids 1-acetyl-β-carboline (**5**) and indole-3-acetic acid (**6**) [16,17]. Saquayamycin B (**4**) displayed potent cytotoxic activity against hepatoma carcinoma HepG-2, SMMC-7721 and plc-prf-5 cell lines, and it caused apoptosis in SMMC-7721 cells.

## 2. Results and Discussion

From 30 L liquid fermentation broth of the strain *Streptomyces* sp. OC1610.4, cultured for 9 days, 4.6 g of EtOAc extract was obtained. After fractionation by column chromatography and preparative HPLC purification, six yellow or brown amorphous powdered-compounds were isolated from the crude EtOAc extract. The major constituent in the extract was firstly purified and whose molecular formula C_43_H_48_O_16_ was established by the HR-ESI-MS *m*/*z* 838.3298 ([M + NH_4_]^+^, calcd for C_43_H_52_NO_16_, 838.3286) and *m*/*z* 843.2842 ([M + Na]^+^, calcd for C_43_H_48_NaO_16_, 843.2840) (Figure S1). Its ^1^H NMR spectrum displayed complex signals including three pairs of aromatic or olefinic protons from δ_H_ 6.06 to 7.91, more than a dozen methylene and methine protons from δ_H_ 1.40 to 5.39 and five methyl groups (Figure S2). The four oxygenated methine proton signals between δ_H_ 5.01 and 5.40 which, through HSQC spectrum, directly attached to the carbons signals at δ_C_ 96.0, 92.8, 92.1 and 72.0 (Figure S3), along with four doublets of methyl groups are the characteristic of four deoxy sugar molecules, one of which probably formed a C-glycoside since its anomeric carbon appeared at δ_C_ 72.0 [18,19]. These data, especially the signals of the deoxy sugar C-glycosidic moiety suggested the structure of angucycline glycoside [1]. Detailed comparison of its ^1^H and ^13^C NMR data with those previously reported in the literature and analysis of the 2D NMR sprectra (Figures S5–S8), led to the identification of this compound as saquayamycin B (**4**) [3,18].

**Figure 2:**
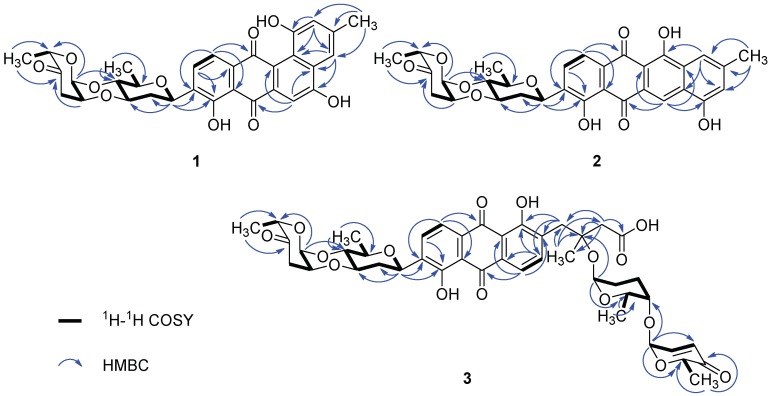
COSY and selected HMBC correlations for **1**–**3**.

Landomycin N (**1**) was a minor constituent of the crude extract. Its molecular formula C_31_H_28_O_10_ was established by the *m*/*z* 561.1753 ([M + H]^+^, calcd for C_31_H_29_O_10_, 561.1761) from HR-ESI-MS. The IR spectrum showed the absorption band of hydroxyl (3203 cm^−1^), carbonyl (1726, 1629 cm^−1^) and aromatic (1607, 1578 cm^−1^) groups. The ^1^H and ^13^C NMR, in combination with APT and HMQC spectra (Figures S11 and S12), revealed the presence of five aromatic protons, seven oxygenated methines, two methylenes and three methyl groups (Table 1). The five aromatic protons at δ_H_ 7.84 (d, *J* = 7.9 Hz), 7.72 (d, *J* = 7.9 Hz), 7.62 (brs), 7.46 (s) and 6.96 (brs), similar to those of urdamycin N4 [4], were assigned to the benz[a]anthraquinone nucleus of angucycline aglycone. The COSY spectrum exhibited the correlations from δ_H_ 7.84 (H-10) to δ_H_ 7.72 (H-11) and from δ_H_ 7.62 (H-2) to δ_H_ 6.96 (H-4) (Figure 2 and Figure S14). The HMBC correlations from δ_H_ 7.84 (H-10) to C-8 (δ_C_ 156.9) and C-11a (δ_C_ 134.7), δ_H_ 7.72 (H-11) to C-7a (δ_C_ 114.1), C-9 (δ_C_ 135.0) and C-12 (δ_C_ 182.6), and δ_H_ 7.46 (H-6) to C-4a (δ_C_ 130.6), C-7 (δ_C_ 188.9) and C-12a (δ_C_ 119.6) supported the presence of anthraquinone nucleus of angucycline aglycone. Although C-12 signal was not observed in the ^13^C NMR spectrum, its chemical shift value was assigned as δ_C_ 182.6 through the correlation from H-11 to this signal in the HMBC spectrum. The presence of the hydroxyl substituent on C-8 on the anthraquinone nucleus was supported by the HMBC correlations from H-10 (δ_H_ 7.84) to C-8 (δ_C_ 156.9), and 8-OH (δ_H_ 12.53) to C-7a (δ_C_ 114.1), C-8 (δ_C_ 156.9) and C-9 (δ_C_ 135.0). The HMBC correlations from CH_3_ (δ_H_ 2.40) to C-2 (δ_C_ 119.4), C-3 (δ_C_ 139.0), C-4 (δ_C_ 114.2), H-2 (δ_H_ 6.96) to C-1 (δ_C_ 155.4), C-4 (δ_C_ 114.2) and C-12b (δ_C_ 122.1), and H-4 (δ_H_ 7.62) to C-2 (δ_C_ 119.4), C-4a (δ_C_ 130.6) and C-12b (δ_C_ 122.1) confirmed the structure of the fourth ring conjugated to anthraquinone nucleus and the attachment of hydroxyl group at C-1 (δ_C_ 155.4) (Figure 2). The chemical shift of C-5 (δ_C_ 166.4) along with the HMBC correlation from H-4 (δ_H_ 7.26) to C-5 suggested the presence of the hydroxyl group at C-5.

**Figure 3:**
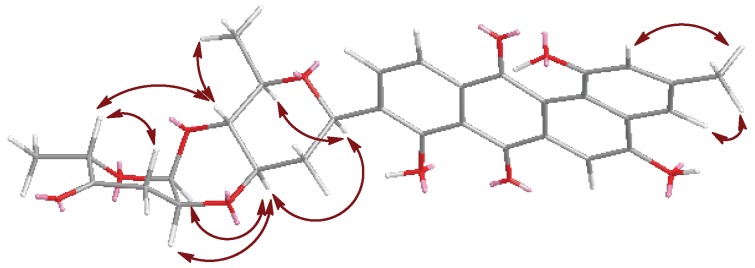
Key NOESY correlations for **1**.

The ^1^H and ^13^C NMR spectra of **1** showed that its aliphatic proton and carbon signals were very similar to those of marangucycline B which has a disaccharide composed of β-d-olivose and α-l-cinerulose B [20]. The observed COSY correlations from H-1A (δ_H_ 4.97) through H-6A (δ_H_ 1.26) confirmed the presence of an olivose (Figure 2). The COSY correlations from H-1B (δ_H_ 5.22) through H-3B (δ_H_ 2.47, 2.90), along with the HMBC correlations from CH_3_-6B (δ_H_ 1.24) to C-4B (δ_C_ 208.7) and C-5B (δ_C_ 76.9), H-1B (δ_H_ 5.22) to C-5B (δ_C_ 76.9), and H-2B (δ_H_ 4.34) to C-4B (δ_C_ 208.7) confirmed the structure of cinerulose B. The linkage of two deoxy sugars was deduced by the HMBC correlations from H-1B to C-4A, and the NOESY correlation between H-2B to H-3A in the most stable conformation obtained by optimizing the molecule to minimized energy by MM2 in ChemBio3D Ultra 14.0 software (Figure 3). The relative configurations of both deoxy sugars were identified as β-d-olivose and α-l-cinerulose B, respectively, by NOESY correlations H-1A/H-5A,3A, H-3A/H-1B,2B, and H-4A/H-6A,5B (Figure 3). Based on the HMBC correlations from H-1A to C-8, H-1A to C-9 and H-1A to C-10, this disaccharide was linked to the aglycone at C-9 through C-1 of β-d-olivose moiety. Thus, the structure of **1** was established and named as landomycin N according to the structural classification code of angucycline initially proposed by Rohr et al. [1] (Figure 1).

**Figure 4:**
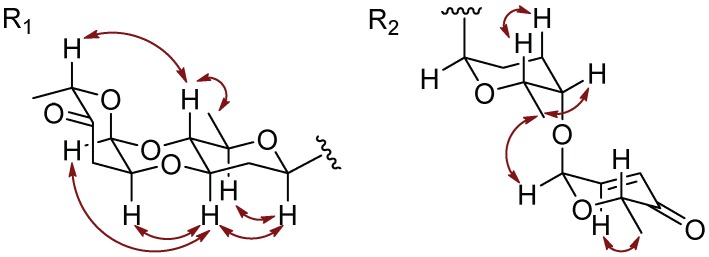
Key NOESY correlations in the sugar moiety of **2** and **3**.

Galtamycin C (**2**) is an isomer of **1**, due to its HRESIMS data *m*/*z* 561.1752 [M + H]^+^ (calcd for C_31_H_29_O_10_, 561.1761). The ^1^H and ^13^C NMR spectra showed that its aliphatic proton and carbon signals were similar to those of **1**, suggesting the presence of the disaccharide α-l-cinerulose B-(1→4, 2→3)-β-d-olivosyl moiety (Table 1). The ^1^H NMR of **2** also showed five aromatic proton signals at δ_H_ 8.39 (s), 7.87 (d, *J* = 7.8 Hz), 7.73 (d, *J* = 7.8 Hz), 7.52 (brs) and 6.95 (brs), where the singlet at δ_H_ 8.39 (s) has higher frequency than the corresponding singlet of **1**. The ^13^C NMR spectrum (Table 1) dispalyed sixteen aromatic carbons with chemical shifts ranging from δ_C_ 108.8 to 162.1 and two quinone carbonyl carbons at δ_C_ 187.3 and 186.3 were similar to those of rearranged linear angucycline glycosides, galtamycinone, grincamycin and grincamycin H [7,21]. Hence, **2** was suggested to possess a linear tetracyclic system. The structure of the compound and the relative configurations of the two deoxysugars were confirmed by COSY, HMBC and NOESY correlations (Figure 2 and Figure 4). Therefore, **2** was named galtamycin C (Figure 1).

Vineomycin D (**3**) was isolated as a yellow powder. Its HR-ESI-MS displayed the quasimolecular ion at *m*/*z* 838.3292 ([M + NH_4_]^+^, calcd for C_43_H_52_NO_16_, 838.3286) and *m*/*z* 843.2838 ([M + Na]^+^, calcd for C_43_H_48_NaO_16_, 843.2840), indicating the same molecular formula (C_43_H_48_O_16_) as saquayamycin B (**4**). Similar to that of saquayamycin B, the ^1^H NMR of **3** also showed two pairs of coupling protons signals at δ_H_ 7.94 (d, *J* = 7.8 Hz, H-10) and 7.80 (d, *J* = 7.8 Hz, H-11), and δ_H_ 7.84 (d, *J* = 7.8 Hz, H-5) and 7.75 (d, *J* = 7.8 Hz, H-6), along with a pair of olefinic protons signals of *α*,*β*-conjugated carbonyl group at δ_H_ 7.03 (dd, *J* = 10.2, 3.5 Hz, H-2D) and 6.02 (d, *J* = 10.2 Hz, H-3D) (Table 1). The ^1^H and ^13^C NMR spectra also revealed the presence of three O-glycosidic anomeric proton and carbon signals at δ_H_ 5.31 (d, *J* = 3.5 Hz)/δ_C_ 96.0 (CH-1D), δ_H_ 5.26 (d, *J* = 2.8 Hz)/δ_C_ 92.2 (CH-1B), and δ_H_ 5.20 (brs)/δ_C_ 92.0 (CH-1C), and one C-glycosidic anomeric proton and carbon signals at δ_H_ 5.01 (brd, *J* = 10.9 Hz)/δ_C_ 72.1 (CH-1A). The most obvious difference in ^13^C NMR spectra of **3** and **4** is the absence of a signal above δ_C_ 200 in **3**, and the presence of a signal at δ_C_ 172.2, characteristic of a carboxylic acid or ester group. Accordingly, **3** was suggested to have a tricyclic system with a side chain, probaly due to the opening of the cyclohexanone ring of saquayamycin B (**4**) [6,7,22]. The skeleton of anthraquinone and the positions of two hydroxyl groups at C-8 and C-12b were confirmed by the HMBC correlations associated with the two pairs of aromatic protons. In HMBC spectrum, the correlations from δ_H_ 7.94 (H-10) to C-8 (δ_C_ 159.6) and C-11a (δ_C_ 133.0), δ_H_ 7.80 (H-11) to C-7a (δ_C_ 116.3), C-9 (δ_C_ 138.8) and C-12 (δ_C_ 189.2), δ_H_ 7.84 (H-5) to C-6a (δ_C_ 132.5) and C-12b (δ_C_ 162.4), δ_H_ 7.75 (H-6) to C-4a (δ_C_ 136.4), C-7 (δ_C_ 189.1) and C-12a (δ_C_ 116.4) were observed (Figure 2). The correlations from the methyl protons at δ_H_ 1.43 (H-13) to C-2 (δ_C_ 44.6), C-3 (δ_C_ 78.0) and C-4 (δ_C_ 39.0), along with the correlations from the methylene protons appearing as a couple of AB system at δ_H_ 2.72 and 2.63 (H-2) to C-1 (δ_C_ 172.1), confirmed the side chain. The linkage between the anthraquinone and side chain was deduced to be at C-4a by the HMBC correlations from methylene protons at δ_H_ 3.23 and 3.19 (H-4) to C-4a (δ_C_ 136.4), C-5 (δ_C_ 140.9) and C-12b (δ_C_ 162.4). The presence of two disaccharides α-L-cinerulose B-(1→4, 2→3)-β-d-olivosyl and α-l-aculose-(1→4)-α-l-rhodinosyl groups were further deduced by COSY, HMBC and NOESY correlations (Figure 2 and Figure 4). The HMBC correlations from H-1A (δ_H_ 5.01) to C-8 (δ_C_ 159.6), C-9 (δ_C_ 138.2) and C-10 (δ_C_ 134.3) suggested that the α-l-cinerulose B-(1→4, 2→3)-β-d-olivosyl group was linked to C-9 through C-1 of d-olivose moiety. The HMBC correlation from H-3A (δ_H_ 5.20) to C-3 (δ_C_ 78.0) indicated that α-l-aculose-(1→4)-α-l-rhodinosyl group was linked to C-3. In general, tricyclic angucyclines are derived from typical angucyclines with the same tetracyclic core structure under acidic conditions [1]. Accordingly, the absolute configuration of C-3 is proposed to be same as that of saquayamycin B (**4**) and other tricyclic angucyclines, e.g., grincamycin B, vineomycin B_2_ and fridamycin D [6,7,22]. Thus, the structure of **3** was established and named vineomycin D (Figure 1).

**Figure 5:**
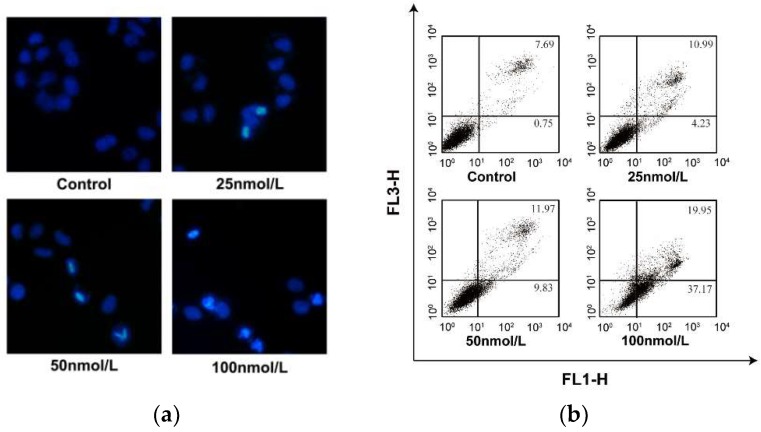
(**a**) Fluorescence micrographs of untreated and saquayamycin B-treated SMMC-7721 cells (24 h) stained with DAPI, Magnification: 100×; (**b**) Quantification of saquayamycin B-induced apoptosis in SMMC-7721 cell using flow cytometric analysis. ** *p* < 0.01 versus saquayamycin B 0 μM group.

A few anguclines, such as saquayamycin B, landomycin E, vineomycin A_1_ etc., have been reported to exhibit remarkable antitumor activity against a series of tumor cell lines [3,7,10]. Though, the distinct in vivo toxicity restricted the further development of these compounds to be clinical drugs. Recently, an atypical angucycline, lomaiviticin A, was reported to be under preclinical evaluation for antitumor treatment due to its prominent cytotoxicity and effects of inducing double-strand breaks in DNA [14,23]. In present work, **1**–**4** were assayed for their cytotoxic activity against normal liver cell LO_2_, hepatoma carcinoma HepG-2, SMMC-7721 and plc-prf-5 cell lines by 3-(4,5-dimethylthiazol-2-yl)-2,5-diphenyltetrazolium bromide (MTT) method (Table 2). At the concentrations of 40 μM, **1**–**3** displayed no cytotoxicity against any of the tested cell lines. Saquayamycin B (**4**) displyed potent cytotoxic activity against HepG-2, SMMC-7721 and plc-prf-5 cells, with IC_50_ values 0.135, 0.033 and 0.244 μM, respectively, which are less than the IC_50_ of doxorubicin. Treatment of SMMC-7721 cells with saquayamycin B at concentrations ranging from 0.025 to 0.100 μM for 24 h, SMMC-7721 cells resulted in chromatin dispersion and formation of apoptotic body in DAPI staining test (Figure 5a). The apoptotic ratio of SMMC-7721 cells was dependent on the concentrations of saquayamycin B (Figure 5b).

## 3. Materials and Methods

### 3.1. General Experimental Procedures

Optical rotations were measured with an Anton Paar MCP 200 polarimeter with a sodium lamp (589 nm) (Anton Paar GmbH, Graz, Austria). UV spectra were obtained on Genesys 10S UV-Vis spectrometer (Thermo Fisher Scientific Ltd, Waltham, MA, USA); IR spectra were recorded with a Nicolet IS5 FT-IR spectrometer (Thermo Fisher Scientific Ltd, Waltham, MA, USA); NMR spectra were recorded on Bruker AVANCE III 500 spectrometer (Bruker Inc., Karlsruhe, Germany). HPLC-MS were acquired on Agilent 1200HPLC/6520QTOFMS (Agilent Technologies Inc., Santa Clara, CA, USA). Semi-preparative HPLC isolation was performed on Agilent 1260 Infinity II (Agilent Technologies Inc., Santa Clara, USA) with an ODS column (YMC-Triart C18, 10 mm × 250 mm, YMC Co. Ltd., Tokyo, Japan). Silica gel (200–300 and 300–400 mesh) used in column chromatography (CC) and silica gel GF_254_ (10–40 µm) used in thin layer chromatography (TLC) were supplied by Qingdao Marine Chemical Factory in China.

### 3.2. Actinomycetes Strain

The intertidal sediment was collected after the tide has ebbed in Xiaoshi Island, Weihai, China in September 2016. The strain OC1610.4 was isolated from this sediment using Gause’s synthetic medium (20 g/L amylogen, 1 g/L KNO_3_, 0.5 g/L NaCl, 0.5 g/L K_2_HPO_4_·H_2_O, 0.5 g/L MgSO_4_·H_2_O, 0.01 g/L FeSO_4_·H_2_O, and 3.0% sea salt) containing potassium dichromate (6 μg/mL) and nalidixic acid (20 μg/mL) as antifungal and antibacterial agents. The procedures of DNA extraction and PCR amplification of 16S rRNA were same as described in reference [24]. The nucleotide sequence of the OC1610.4 strain was sequenced at the Shanghai Sangon Biotech Co., China, and deposited at GenBank (Accession no. MK045847). Voucher strain (No. OC1610.4) was deposited at Laboratory of Natural Products Chemistry, Department of Pharmacy, Shandong University at Weihai.

### 3.3. Fermentation, Extraction and Isolation

The spore and mycelia suspension of strain OC1610.4 was inoculated in Erlenmeyer flasks (500 mL) each of which contains 100 mL S-medium (10 g/L glucose, 4 g/L yeast extract, 4 g/L K_2_HPO_4_, 2 g/L KH_2_PO_4_, 0.5 g/L MgSO_4_·7H_2_O, and 3.0% sea salt). Total 30 L medium was shaking-cultured at 140 rpm and 28 °C for 9 days. The fermentation broth including mycelia was extracted with equal volume of EtOAc five times to give 4.6 g crude extract. The extract was subjected to silica gel CC (60 g, 200–300 mesh) eluting with n-hexane-acetone (10:1, 5:1, 2:1 and acetone) to give four fractions F_1_–F_4_. Part (72 mg) of fraction F_1_ (n-hexane-acetone 10:1) was isolated by semi-preparative HPLC eluting with CH_3_OH-H_2_O (70:30, *v*/*v*) to give **5** (5.6 mg). Fraction F_2_ (n-hexane-acetone 5:1, 267 mg) was further purified by silica gel CC (1 g, 300–400 mesh) eluting with n-hexane-acetone (10:1) to give sub-fractions F_2a_ and F_2b_. Sub-fractions F_2a_ (67 mg) was purified by semi-preparative HPLC eluting with CH_3_OH-H_2_O (38:62, *v*/*v*) to give **6** (4.6 mg). The sub-fractions F_2b_ (26 mg) was a mixture presenting two brown spots on TLC, and was isolated by semi-preparative HPLC eluting with CH_3_CN-H_2_O (70:30, *v*/*v*) to give **1** (4.2 mg) and **2** (3.4 mg). Fraction F_3_ (n-hexane-acetone 2:1, 670 mg) was subjected to a silica gel CC (10 g, 200–300 mesh) eluting with CH_3_Cl-CH_3_OH (20:1) to give two subfractions F_3a_ and F_3b_. From F_3a_ (220 mg), compound **4** (18 mg) was purified using a low pressure silica gel CC (1 g, 300–400 mesh) eluting with n-hexane-acetone (4:1). Subfractions F_3b_ (67 mg) was isolated by semi-preparative HPLC eluting with CH_3_CN-H_2_O (65:35, *v*/*v*) to give **3** (5 mg).

Landomycin N (**1**): brown amorphous powder; ${\left[\alpha \right]}_{D}^{25}$ +92 (*c* 0.002, MeOH); UV (MeOH) λ_max_ (log ε) 225 (2.99), 327 (2.65) nm; IR (KBr) *ν*_max_ 3203, 2974, 2916, 1726, 1629, 1607, 1578, 1433, 1295, 1111, 1075, 852, 791 cm^−1^; ^1^H NMR (500 MHz, DMSO-*d*_6_) and ^13^C NMR (125 MHz, DMSO-*d*_6_) data, Table 1; HR-ESI-MS *m*/*z* 561.1753 ([M + H]^+^, calcd for C_31_H_29_O_10_, 561.1761).

Galtamycin C (**2**): reddish-brown amorphous powder; ${\left[\alpha \right]}_{D}^{25}$ +285 (*c* 0.003, MeOH); UV (MeOH) λ_max_ (log ε) 265 (2.40), 340 (2.07) nm; IR (KBr) *ν*_max_ 3383, 2917, 2879, 1727, 1657, 1608, 1584, 1525, 1471, 1286, 1247, 1108, 1017, 872, 836, 716 cm^−1^; ^1^H NMR (500 MHz, DMSO-*d*_6_) and ^13^C NMR (125 MHz, DMSO-*d*_6_) data, Table 1; HR-ESI-MS *m*/*z* 561.1752 ([M + H]^+^, calcd for C_31_H_29_O_10_, 561.1761).

Vineomycin D (**3**): yellow amorphous powder; ${\left[\alpha \right]}_{D}^{25}$ +69 (*c* 0.050, MeOH); UV (MeOH) λ_max_ (log ε) 230 (3.56), 259 (3.28), 295 (2.83) nm; IR (KBr) *ν*_max_ 3557, 2978, 2935, 1731, 1702, 1625, 1581, 1431, 1259, 1080, 1014, 899, 808 cm^−1^; ^1^H NMR (500 MHz, acetone-*d*_6_) and ^13^C NMR (125 MHz, acetone-*d*_6_) data, Table 1; HR-ESI-MS *m*/*z* 838.3292 ([M + NH_4_]^+^, calcd for C_43_H_52_NO_16_, 838.3286) and *m*/*z* 843.2838 ([M + Na]^+^, calcd for C_43_H_48_NaO_16_, 843.2840).

### 3.4. Cytotoxicity Assays, DAPI Staining Test and Flow Cytometric Analysis

The cytotoxicity evaluations of **1**–**4** against normal liver cell and hepatoma carcinoma cells were carried out using the 3-(4,5-dimethylthiazole-2-yl)-2,5-diphenyltetrazolium bromide (MTT) assay. Doxorubicin was used as positive control drug and deionized H_2_O with the same DMSO concentration was used as parallel control. DAPI staining test was employed to qualitatively observe apoptosis, and the apoptotic ratio was measured by flow cytometric analysis (Becton Dickinson FACScan, San Jose, CA, USA). These tests were conducted using the methods as previously described [25,26].

## 4. Conclusions

Four angucycline glycosides including landomycin N (**1**), galtamycin C (**2**), vineomycin D (**3**) and saquayamycin (**4**), along with two alkaloids 1-acetyl-β-carboline (**5**) and indole-3-acetic acid (**6**), were isolated from the fermentation broth of strain *Streptomyces* sp. OC1610.4, obtained from the intertidal sediment. Galtamycin C (**2**) and vineomycin D (**3**) are rearranged angucycline derivatives respectively possessing a linear tetracyclic and a tricyclic framework of angucycline. Vineomycin D (**3**) and saquayamycin B (**4**) are isomers, comprising the same two disaccharides in the structures. Among the isolated angucycline glycosides, saquayamycin B (**4**) displayed the most potent cytotoxic activity against hepatoma carcinoma HepG-2, SMMC-7721 and plc-prf-5 cells. Although saquayamycin B was shown to induce an apoptosis in SMMC-7721 cell, its antineoplastic mechanism needs to be further investigated.

## Figures and Tables

**Table 1:** The ^1^H and ^13^C NMR data of **1**–**3** (500 MHz and 125 MHz) ^a^.

No.		1 ^b^		2 ^b^		3 ^c^
	δC type	δH, mult (*J* in Hz)	δC type	δH, mult (*J* in Hz)	δC type	δH, mult (*J* in Hz)
1	155.4 C	-	155.9 C	-	172.1 C	-
2	119.4 CH	6.96, brs	116.2 CH	6.95, brs	44.6 CH_2_	2.63, d (15.0) 2.72, d (15.0)
3	139.0 C	-	141.8 C	-	78.0 C	-
4	114.2 CH	7.26, brs	114.2 CH	7.52, brs	39.0 CH_2_	3.19, d (13.4) 3.23, d (13.4)
4a	130.6 C	-	128.2 C	-	136.4 C	-
5	166.4 C	-	124.1 C	-	140.9 CH	7.84, d (7.8)
6	106.4 CH	7.46, s	116.7 CH	8.39, s	119.2 CH	7.75, d (7.8)
6a	137.3 C	-	125.1 C	-	132.5 C	-
7	188.9 C	-	187.3 C	-	189.1 C	-
7a	114.1 C	-	116.2 C	-	116.3 C	-
8	156.9 C	-	158.4 C	-	159.6 C	-
9	135.0 C	-	136.3 C	-	138.8 C	-
10	133.7 CH	7.84, d (7.9)	133.2 CH	7.87, d (7.8)	134.3 CH	7.94, d (7.8)
11	119.6 CH	7.72, d (7.9)	118.4 CH	7.73, d (7.8)	119.9 CH	7.80, d (7.8)
11a	134.7 C	-	132.4 C	-	133.0 C	-
12	182.6 C	-	186.3 C	-	189.2 C	-
12a	119.6 C	-	108.8 C	-	116.4 C	-
12b	122.1 C	-	162.1 C	-	162.4 C	-
13	20.9 CH_3_	2.40, s	21.9 CH_3_	2.40, s	23.5 CH_3_	1.43, s
OH	-	12.53, brs	-	14.40, brs	-	13.14, brs
OH	-	12.08, brs	-	13.41, brs	-	13.10, brs
OH	-		-	10.92, brs	-	
**Sugar A, β-d-olivose**						
1A	70.4 CH	4.97, brd (10.5)	70.5 CH	4.96, brd (10.8)	72.1 CH	5.01, brd (10.9)
2A	35.9 CH_2_	1.63, ddd (11.6, 11.6, 10.5) 2.22, m	35.8 CH_2_	1.61, ddd (11.7, 11.7, 10.8) 2.24, m	37.4 CH_2_	1.60, ddd (11.6, 11.6, 10.9) 2.40, m
3A	75.7 CH	3.85, ddd (11.6, 9.0, 4.4)	75.7 CH	3.86, ddd (11.7, 9.0, 4.3)	77.4 CH	3.88, ddd (11.6, 8.9, 4.4)
4A	73.6 CH	3.51, dd (9.0, 9.0)	73.6 CH	3.51, dd (9.0, 9.0)	75.1 CH	3.58, dd (8.9, 8.9)
5A	73.5 CH	3.59, m	73.5 CH	3.60, m	75.1 CH	3.62, m
6A	17.4 CH_3_	1.26, d (6.0)	17.4 CH_3_	1.27, d (6.0)	17.9 CH_3_	1.34, d (5.8)
**Sugar B, α-l-cinerulose B**						
1B	90.5 CH	5.22, d (2.6)	90.2 CH	5.23, d (2.4)	92.2 CH	5.26, d (2.8)
2B	70.8 CH	4.34, m	70.8 CH	4.35, m	72.3 CH	4.33, m
3B	39.6 CH_2_	2.47, dd (17.4, 2.6) 2.90, dd (17.4, 2.6)	39.8 CH_2_	2.47, dd (17.3, 3.4) 2.91, dd (17.4, 2.6)	40.6 CH_2_	2.53, dd (17.3, 3.6) 2.84, dd (17.3, 2.7)
4B	208.7 C	-	208.7 C	-	208.5 C	-
5B	76.9 CH	4.72, q (6.6)	76.9 CH	4.72, q (6.6)	78.2 CH	4.76, q (6.8)
6B	16.0 CH_3_	1.24, d (6.6)	16.0 CH_3_	1.25, d (6.6)	16.5 CH_3_	1.26, d (6.8)
**Sugar C, α-l-rhodinose**						
1C					92.0 CH	5.20, brs
2C					26.2 CH_2_	1.40, m 1.95, m
3C					25.3 CH_2_	1.90, m 2.10, m
4C					77.4	3.65, m
5C					67.0 CH	4.09, m
6C					17.5 CH_3_	1.10, d (6.6)
**Sugar D, α-l-aculose**						
1D					96.0 CH	5.31, d (3.5)
2D					145.2 CH	7.03, dd (10.2, 3.5)
3D					127.2 CH	6.02, d (10.2)
4D					197.3 C	-
5D					71.0 CH	4.56, q (6.8)
6D					15.5 CH_3_	1.27, d (6.8)

^a^ Residual signals of solvent as reference. ^b^ Measured in DMSO-*d*_6_. ^c^ Measured in acetone-*d*_6_.

**Table 2:** Cytotoxicity of **1**–**4** against LO_2_, HepG-2, SMMC-7721 and plc-prf-5 cells (IC_50_, μM).

Compounds	Cell Lines			
	LO_2_	HepG-2	SMMC-7721	plc-prf-5
**1**	>40	>40	>40	>40
**2**	>40	>40	>40	>40
**3**	>40	>40	>40	>40
**4**	0.343 ± 0.081	0.135 ± 0.056	0.033 ± 0.005	0.244 ± 0.001
Doxorubicin	2.26 ± 0.16	0.919 ± 0.599	0.706 ± 0.004	1.03 ± 0.99

## Supplementary Materials

The following are available online at http://www.mdpi.com/1660-3397/16/12/470/s1: This section includes the HR-ESI-MS, 1D and 2D NMR spectra for compounds **1**–**4**. Figures S1–S8: HR-ESI-MS, 1D and 2D NMR spectra of saquayamycin B (**4**); Figures S9–S15: HR-ESI-MS, 1D and 2D NMR spectra of landomycin N (**1**); Figures S16–S22: HR-ESI-MS, 1D and 2D NMR spectra of galtamycin C (**2**); Figures S23–S29: HR-ESI-MS, 1D and 2D NMR spectra of vineomycin D (**3**).
