# Supplementary material for: Angucycline Glycosides from an Intertidal Sediments Strain Streptomyces sp. and Their Cytotoxic Activity against Hepatoma Carcinoma Cells
Source: Mar Drugs. 2018 Nov 27;16(12):470. doi: 10.3390/md16120470 (PMC6315490; doi:10.3390/md16120470)

# Supplementary Materials

# Angucycline Glycosides from an Intertidal Sediments Strain *Streptomyces* sp. and Their Cytotoxic Activity against Hepatoma Carcinoma Cells

**Aihong Peng[1,2], Xinying Qu[1], Fangyuan Liu[1], Xia Li[1], Erwei Li[2,*] and Weidong Xie[1,*]**

[1] Department of Pharmacy, College of Marine Science, Shandong University at Weihai, Weihai 264209, China; e-mails: pengahsdu@163.com (A.P.); quxinying321@163.com (X.Q.); fangyuan617@outlook.com (F.L.); xiali@sdu.edu.cn (X.L.)
[2] State Key Laboratory of Mycology, Institute of Microbiology, Chinese Academy of Sciences, Beijing 100101, China

* Correspondence: wdxie@sdu.edu.cn (W.X.); liew@im.ac.cn (E.L.); Tel.: +86-631-568-8303 (W.X.); +86-10-6480-6141 (E.L.)

# Contents

**Figure S1**. HR-ESI-MS of saquayamycin B (**4**).
**Figure S2**. $^{1}$H-NMR spectrum (500 MHz, acetone-$d_6$) of saquayamycin B (**4**).
**Figure S3**. $^{13}$C-NMR spectrum (125 MHz, acetone-$d_6$) of saquayamycin B (**4**).
**Figure S4**. DEPT-135 spectrum (125 MHz, acetone-$d_6$) of saquayamycin B (**4**).
**Figure S5**. HMQC spectrum (500 MHz, acetone-$d_6$) of saquayamycin B (**4**).
**Figure S6**. HMBC spectrum (500 MHz, acetone-$d_6$) of saquayamycin B (**4**).
**Figure S7**. $^{1}$H-$^{1}$H COSY spectrum (500 MHz, acetone-$d_6$) of saquayamycin B (**4**).
**Figure S8**. NOESY spectrum (500 MHz, acetone-$d_6$) of saquayamycin B (**4**).
**Figure S9**. HR-ESI-MS of **1**.
**Figure S10**. $^{1}$H-NMR spectrum (500 MHz, DMSO-$d_6$) of **1**.
**Figure S11**. $^{13}$C-NMR (APT) spectrum (125 MHz, DMSO-$d_6$) of **1**.
**Figure S12**. HMQC spectrum (500 MHz, DMSO-$d_6$) of **1**.
**Figure S13**. HMBC spectrum (500 MHz, DMSO-$d_6$) of **1**.
**Figure S14**. $^{1}$H-$^{1}$H COSY spectrum (500 MHz, DMSO-$d_6$) of **1**.
**Figure S15**. NOESY spectrum (500 MHz, DMSO-$d_6$) of **1**.
**Figure S16**. HR-ESI-MS of **2**.
**Figure S17**. $^{1}$H-NMR spectrum (500 MHz, DMSO-$d_6$) of **2**.
**Figure S18**. $^{13}$C-NMR (APT) spectrum (125 MHz, DMSO-$d_6$) of **2**.
**Figure S19**. HMQC spectrum (500 MHz, DMSO-$d_6$) of **2**.
**Figure S20**. HMBC spectrum (500 MHz, DMSO-$d_6$) of **2**.
**Figure S21**. $^{1}$H-$^{1}$H COSY spectrum (500 MHz, DMSO-$d_6$) of **2**.
**Figure S22**. NOESY spectrum (500 MHz, DMSO-$d_6$) of **2**.
**Figure S23**. HR-ESI-MS of **3**.
**Figure S24**. $^{1}$H-NMR spectrum (500 MHz, acetone-$d_6$) of **3**.
**Figure S25**. $^{13}$C-NMR (APT) spectrum (125 MHz, acetone-$d_6$) of **3**.
**Figure S26**. HMQC spectrum (500 MHz, acetone-$d_6$) of **3**.
**Figure S27**. HMBC spectrum (500 MHz, acetone-$d_6$) of **3**.
**Figure S28**. $^{1}$H-$^{1}$H COSY spectrum (500 MHz, acetone-$d_6$) of **3**.
**Figure S29**. NOESY spectrum (500 MHz, acetone-$d_6$) of **3**.

**Figure S1**. HR-ESI-MS of saquayamycin B (**4**).

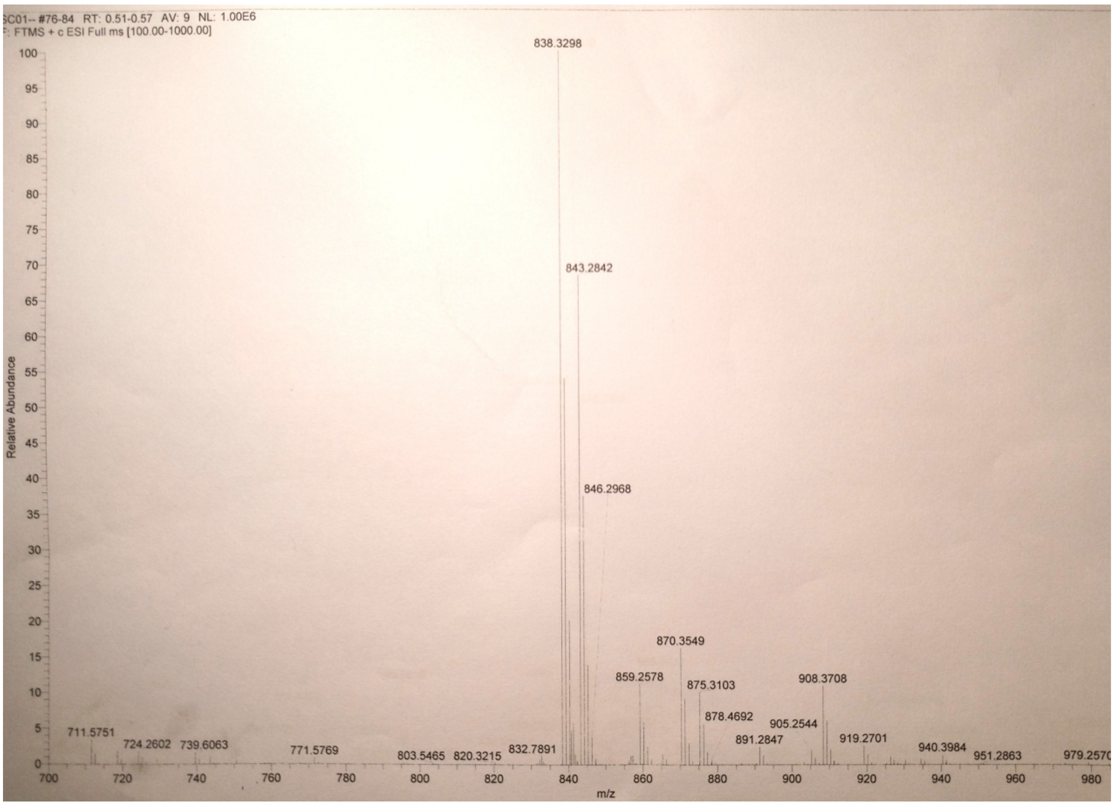


**Figure S2**. $^{1}$H-NMR spectrum (500 MHz, acetone-$d_6$) of saquayamycin B (**4**).

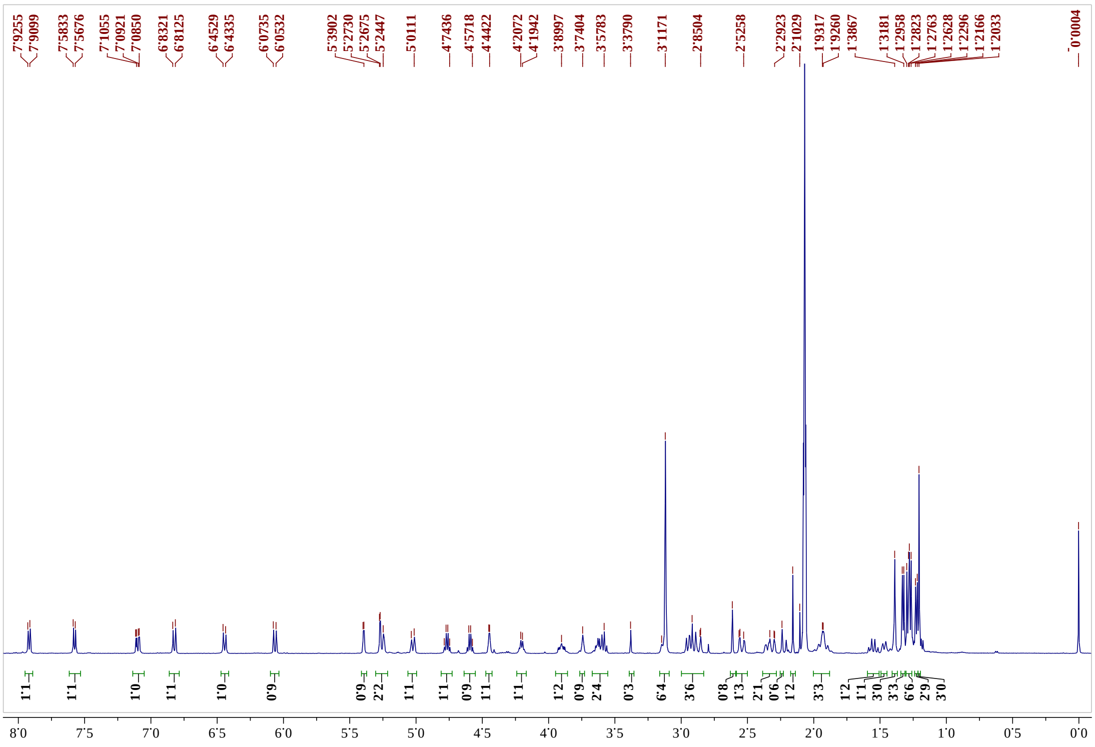

**Figure S3**. $^{13}$C-NMR spectrum (125 MHz, acetone-$d_6$) of saquayamycin B (**4**).

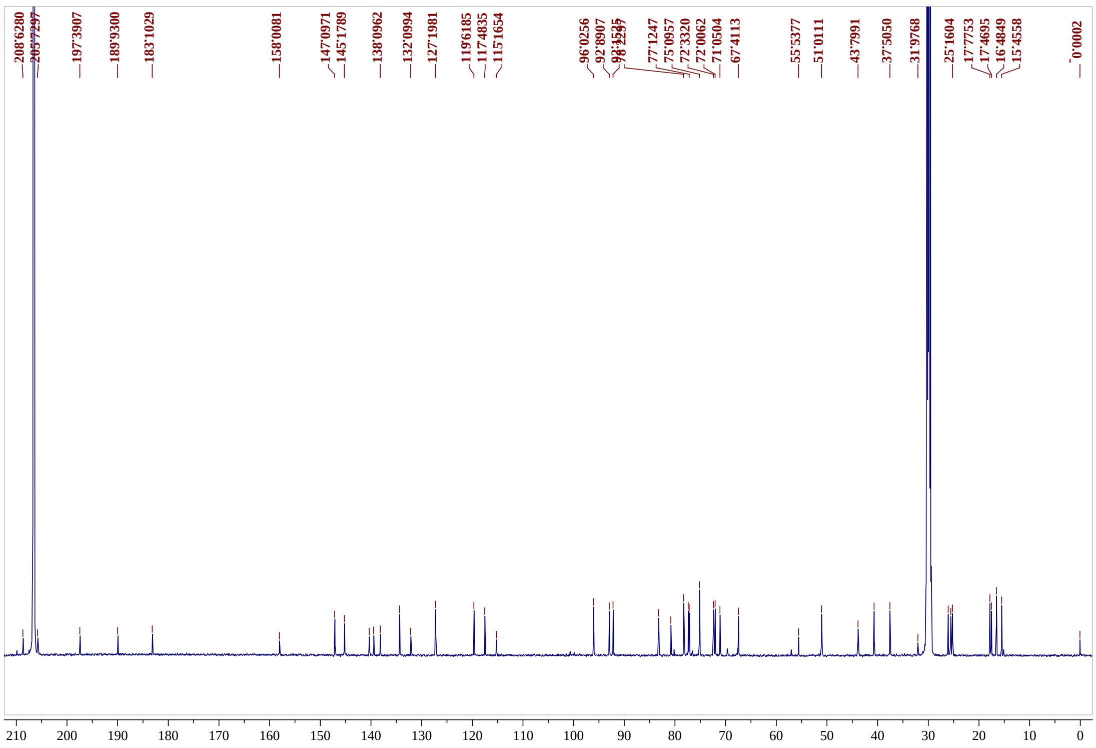


**Figure S4**. DEPT-135 spectrum (125 MHz, acetone-$d_6$) of saquayamycin B (**4**).

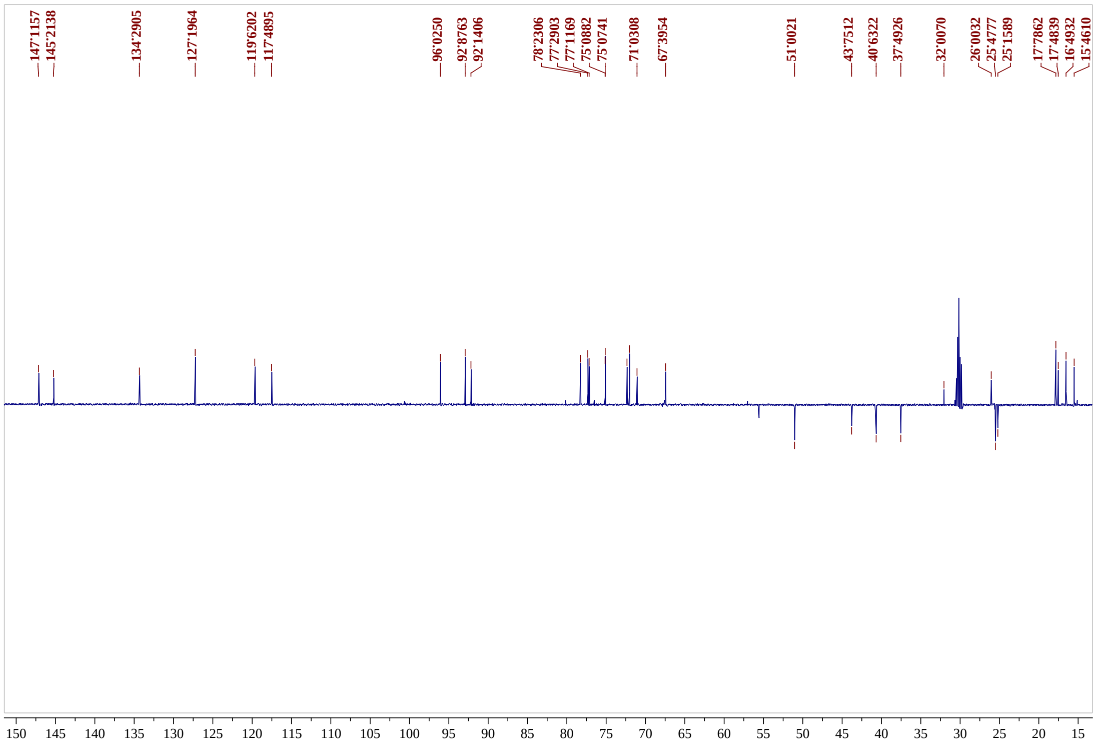

**Figure S5**. HMQC spectrum (500 MHz, acetone-$d_6$) of saquayamycin B (**4**).

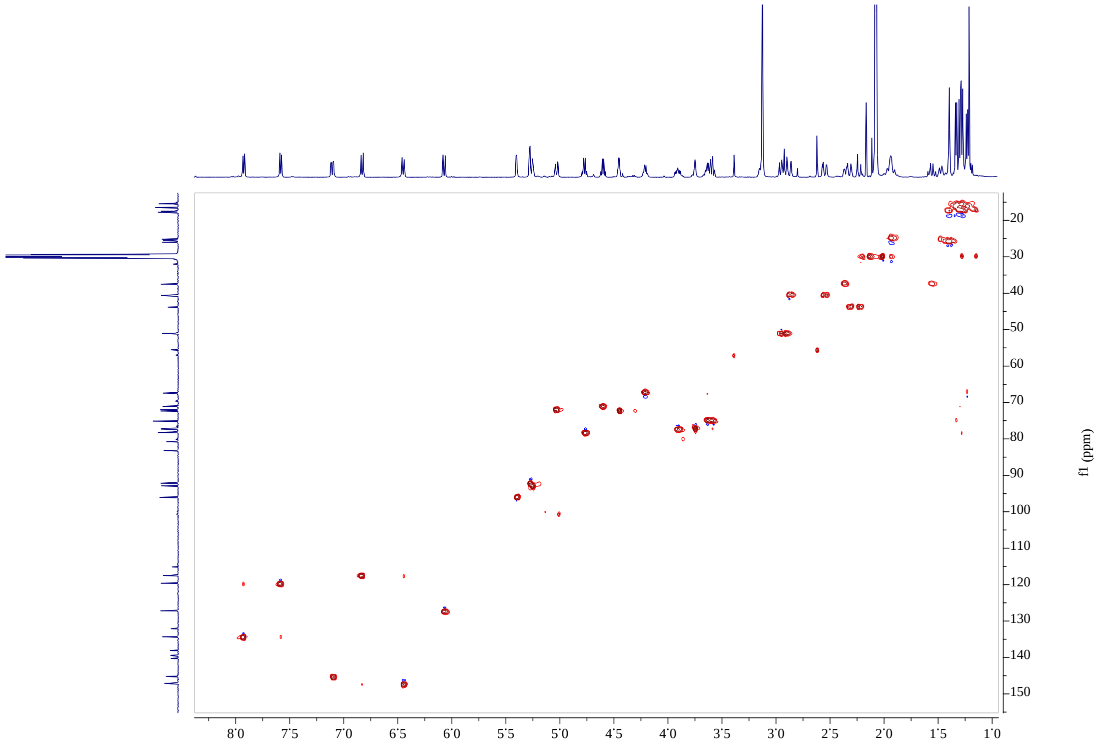


**Figure S6**. HMBC spectrum (500 MHz, acetone-$d_6$) of saquayamycin B (**4**).

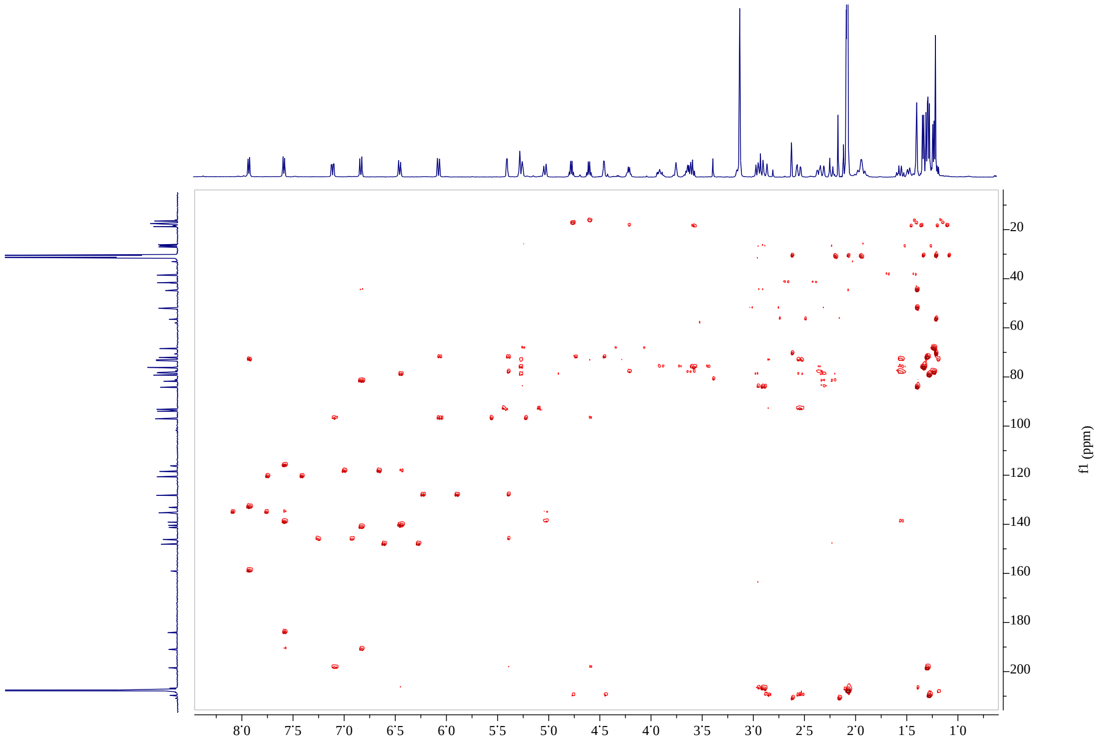

**Figure S7**. $^{1}H$-$^{1}H$ COSY spectrum (500 MHz, acetone-$d_6$) of saquayamycin B (**4**).

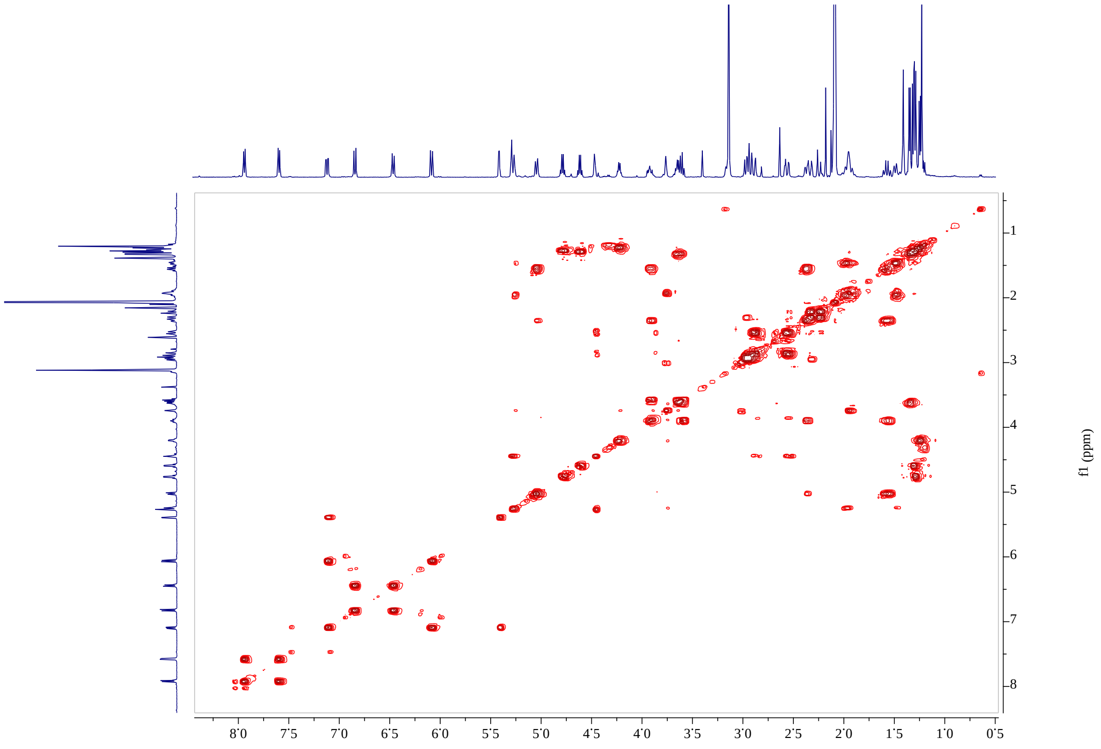


**Figure S8**. NOESY spectrum (500 MHz, acetone-$d_6$) of saquayamycin B (**4**).

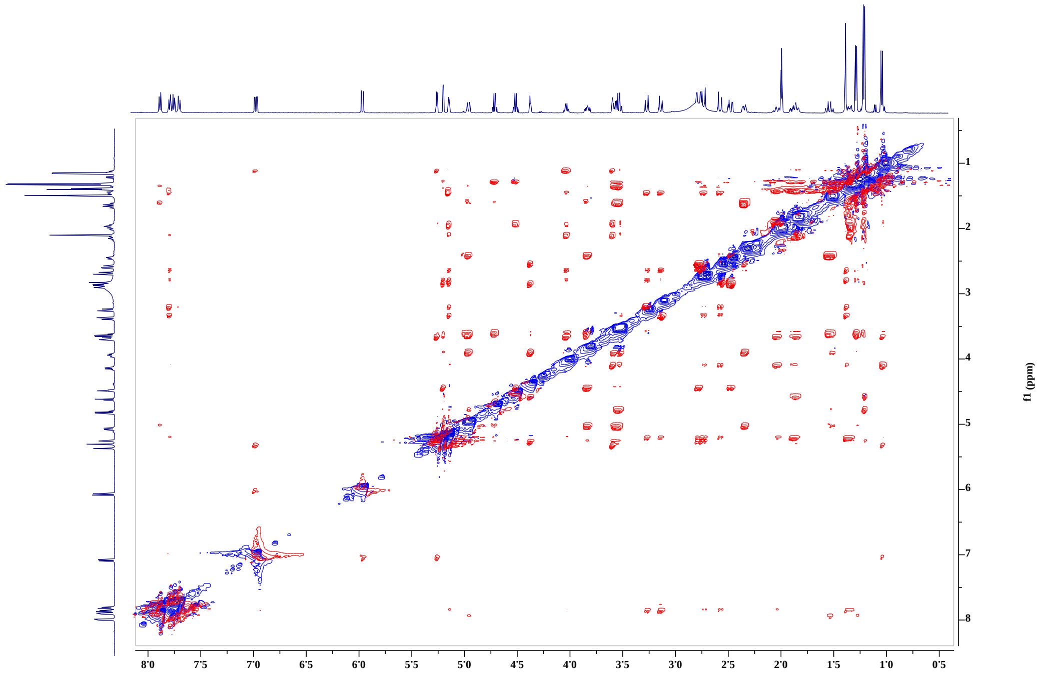

**Figure S9**. HR-ESI-MS of **1**.

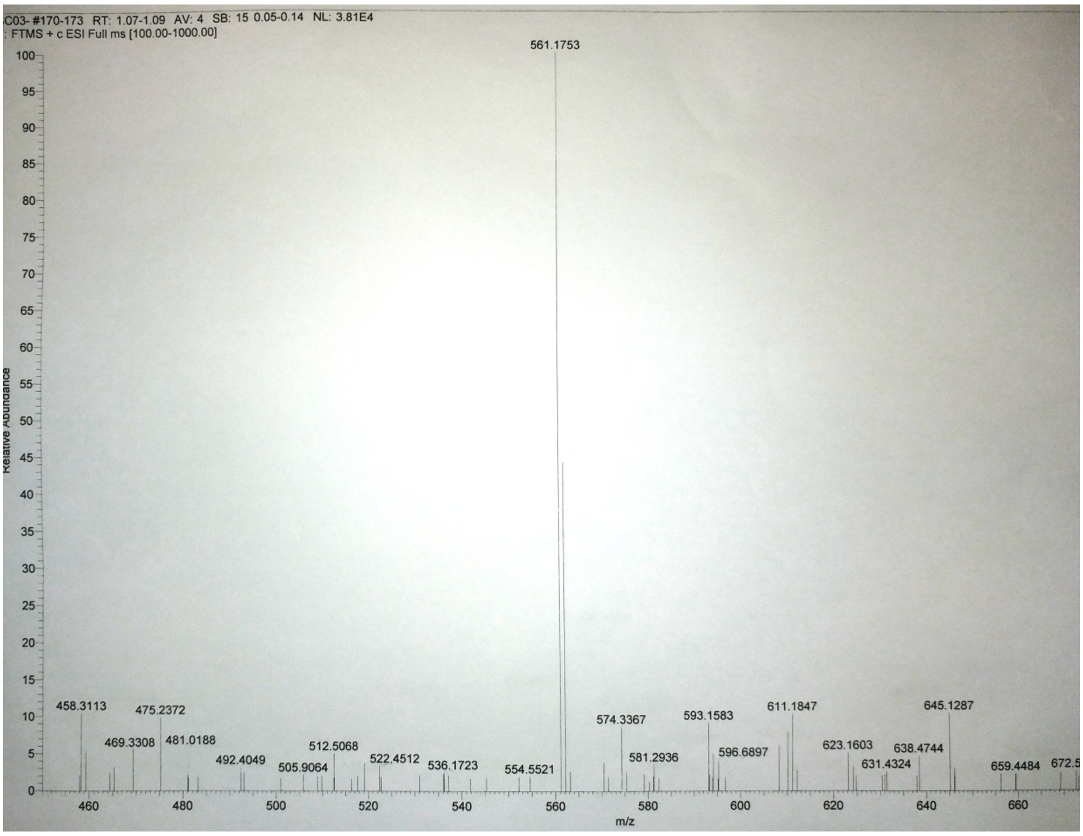


**Figure S10**. $^{1}$H-NMR spectrum (500 MHz, DMSO-$d_6$) of **1**.

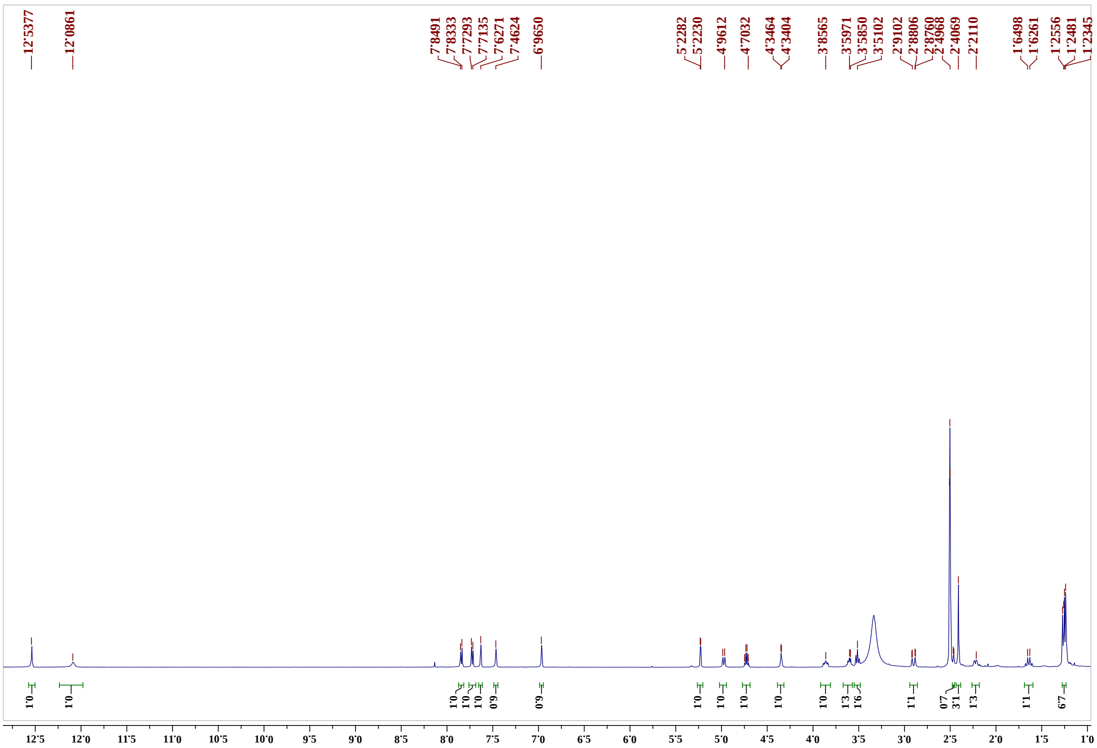

**Figure S11**. $^{13}$C-NMR (APT) spectrum (125 MHz, DMSO-$d_6$) of **1**.

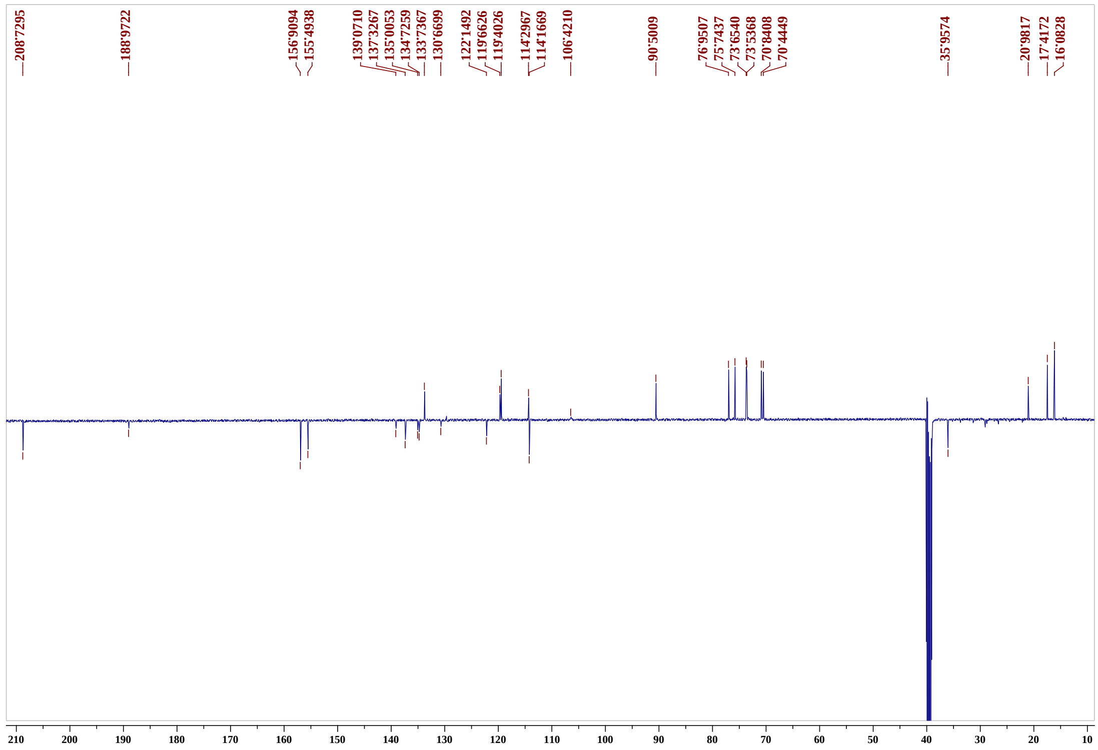

**Figure S12**. HMQC spectrum (500 MHz, DMSO-$d_6$) of **1**.

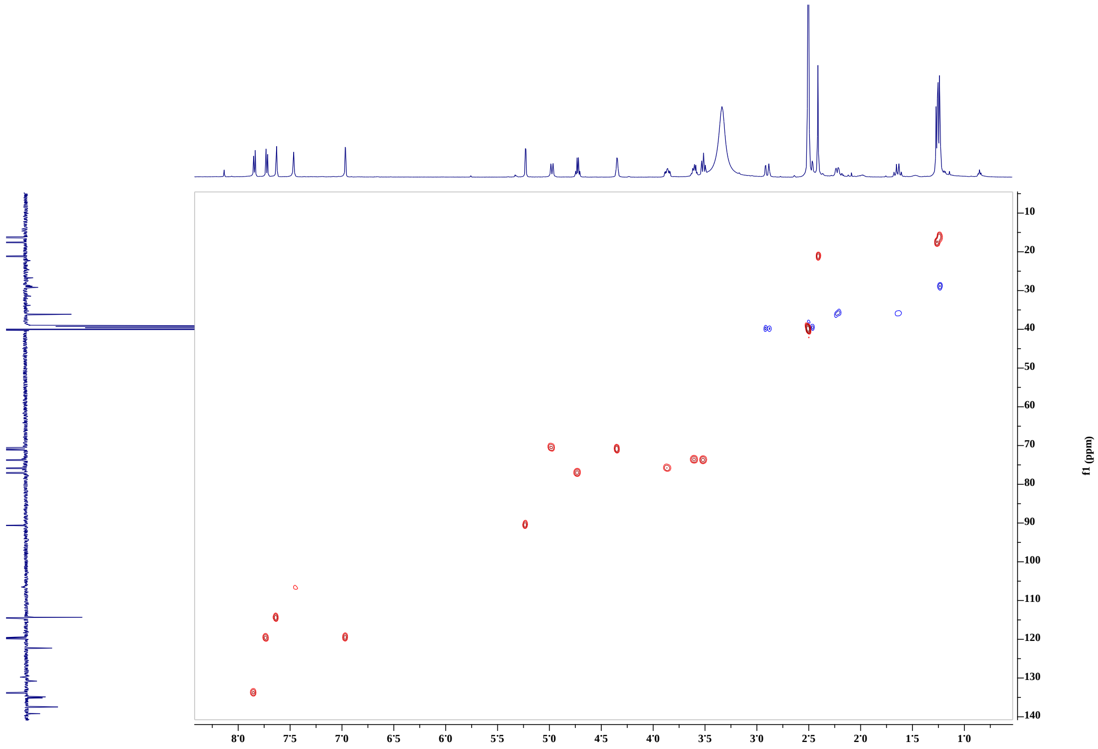

**Figure S13**. HMBC spectrum (500 MHz, DMSO-$d_6$) of **1**.

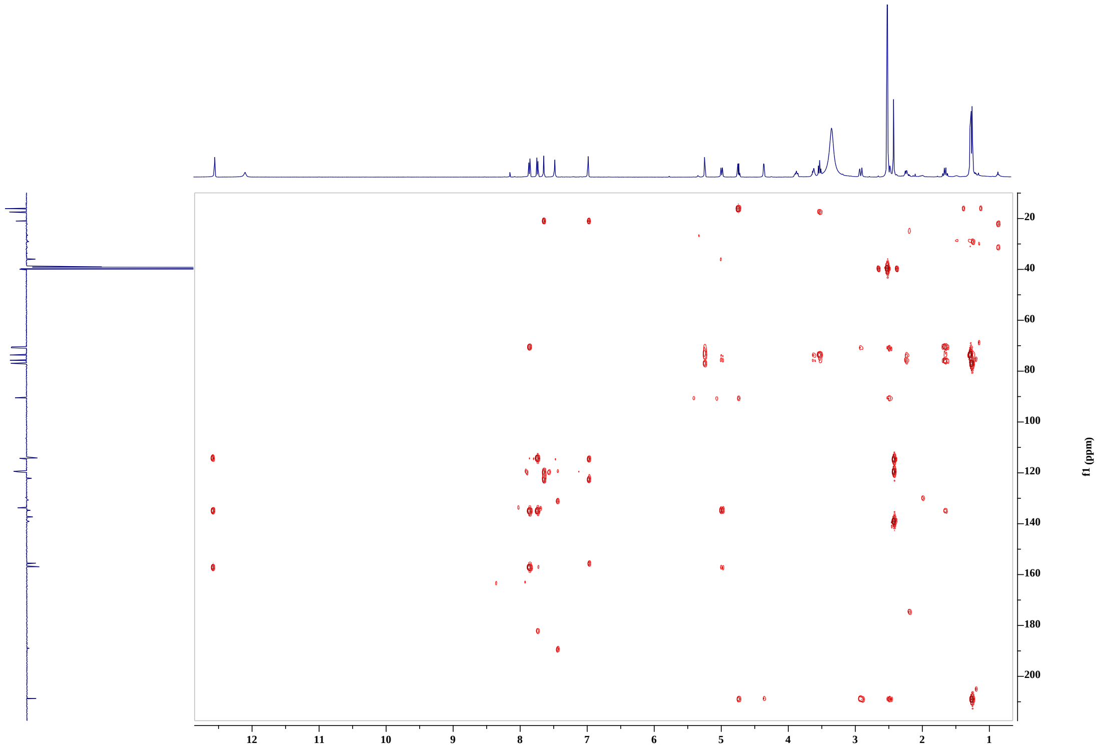

**Figure S14**. $^1H$-$^1H$ COSY spectrum (500 MHz, DMSO-$d_6$) of **1**.

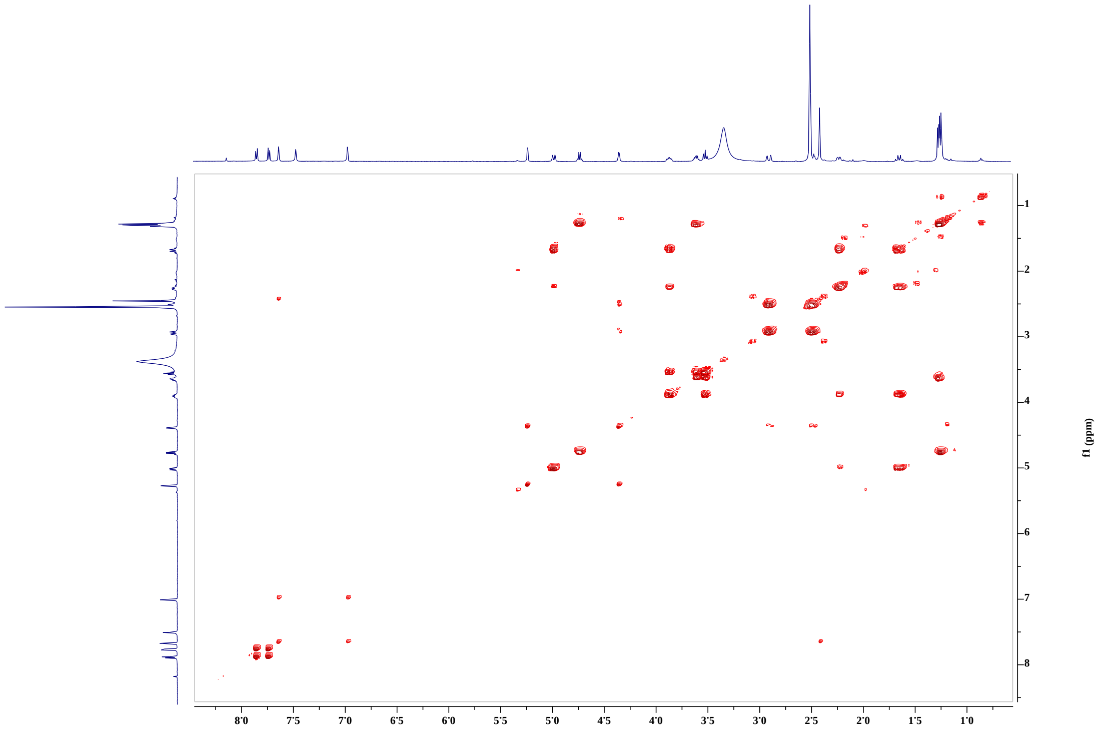

**Figure S15**. NOESY spectrum (500 MHz, DMSO-$d_6$) of **1**.

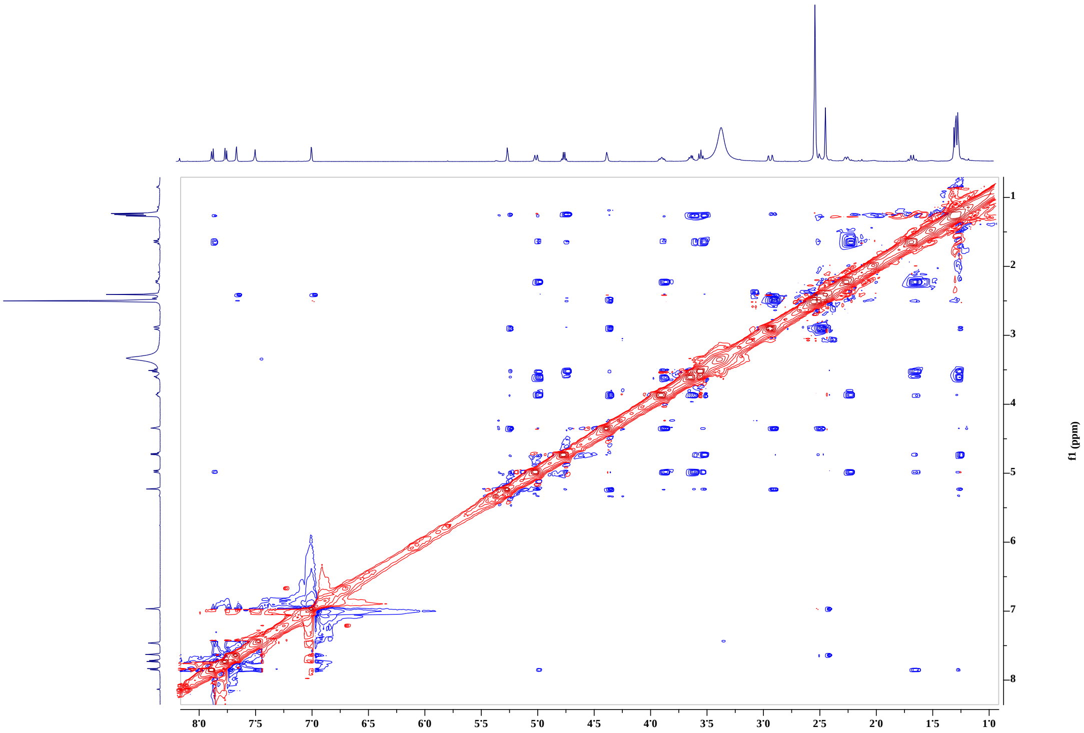


**Figure S16**. HR-ESI-MS of **2**.

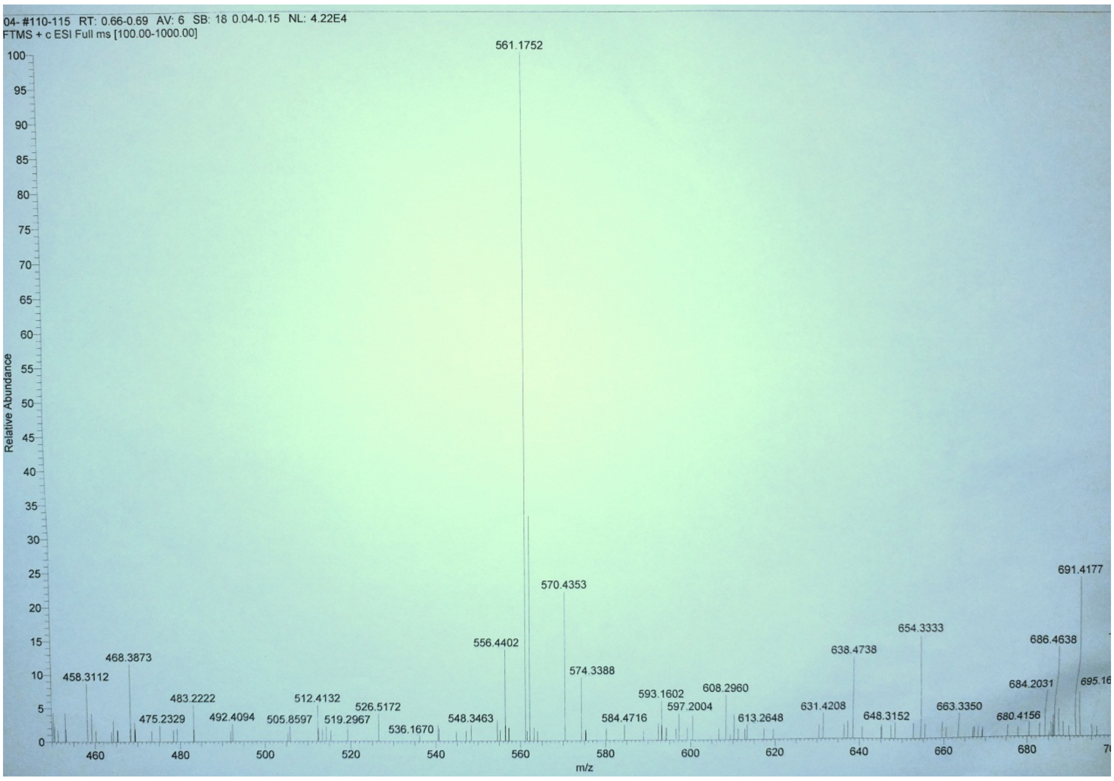

**Figure S17**. $^{1}$H-NMR spectrum (500 MHz, DMSO-d$_6$) of **2**.

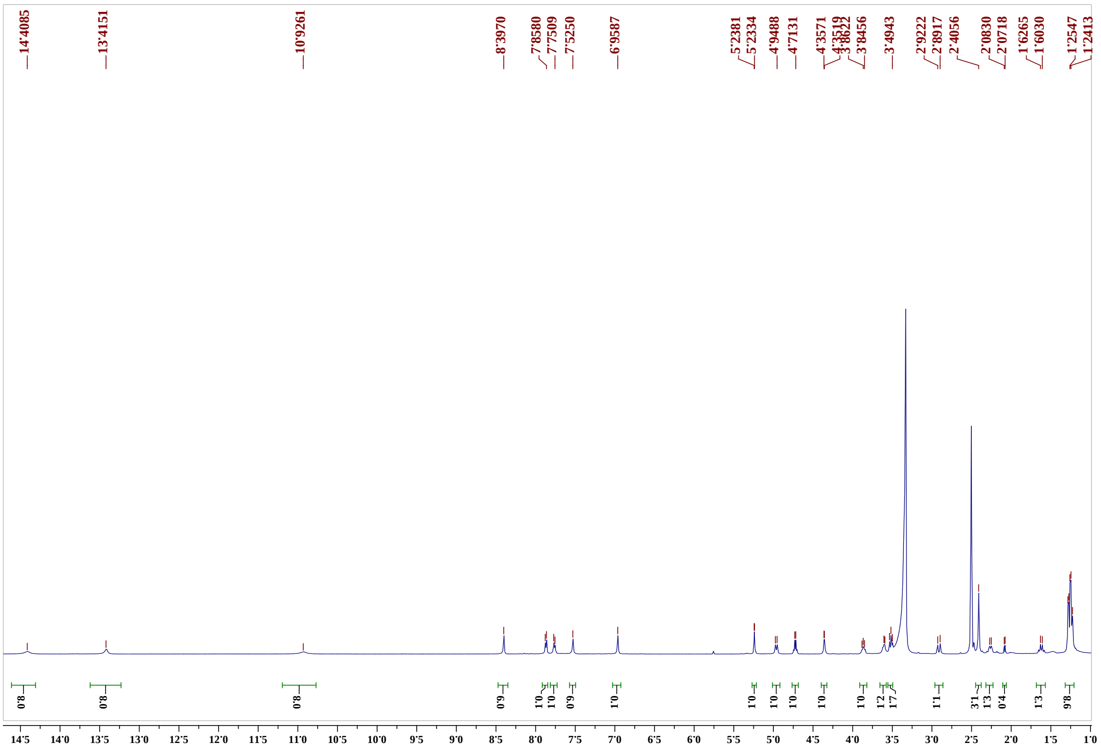

**Figure S18**. $^{13}$C-NMR (APT) spectrum (125 MHz, DMSO-d$_6$) of **2**.

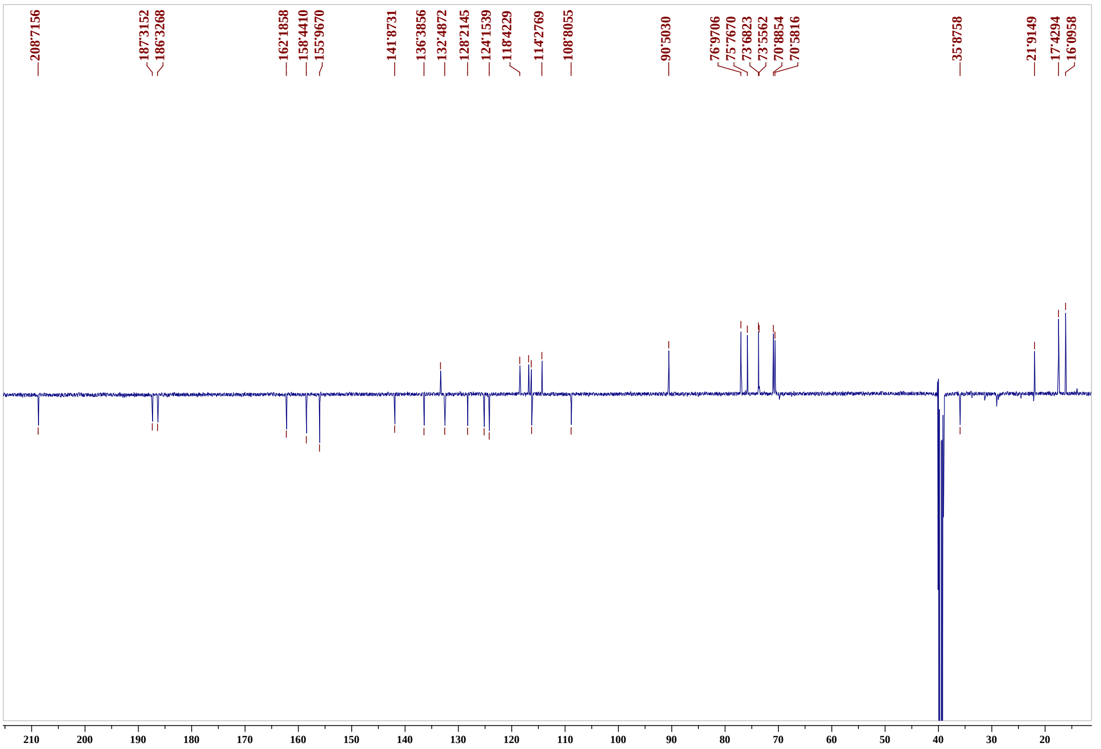

**Figure S19**. HMQC spectrum (500 MHz, DMSO-$d_6$) of **2**.

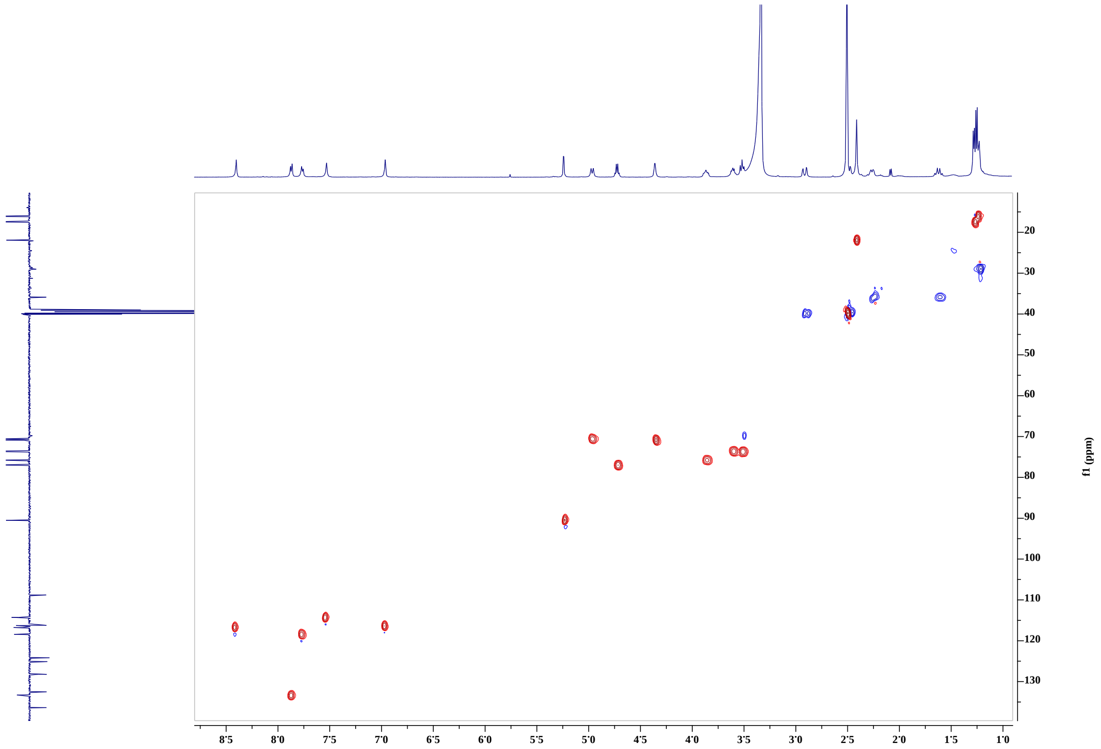


**Figure S20**. HMBC spectrum (500 MHz, DMSO-$d_6$) of **2**.

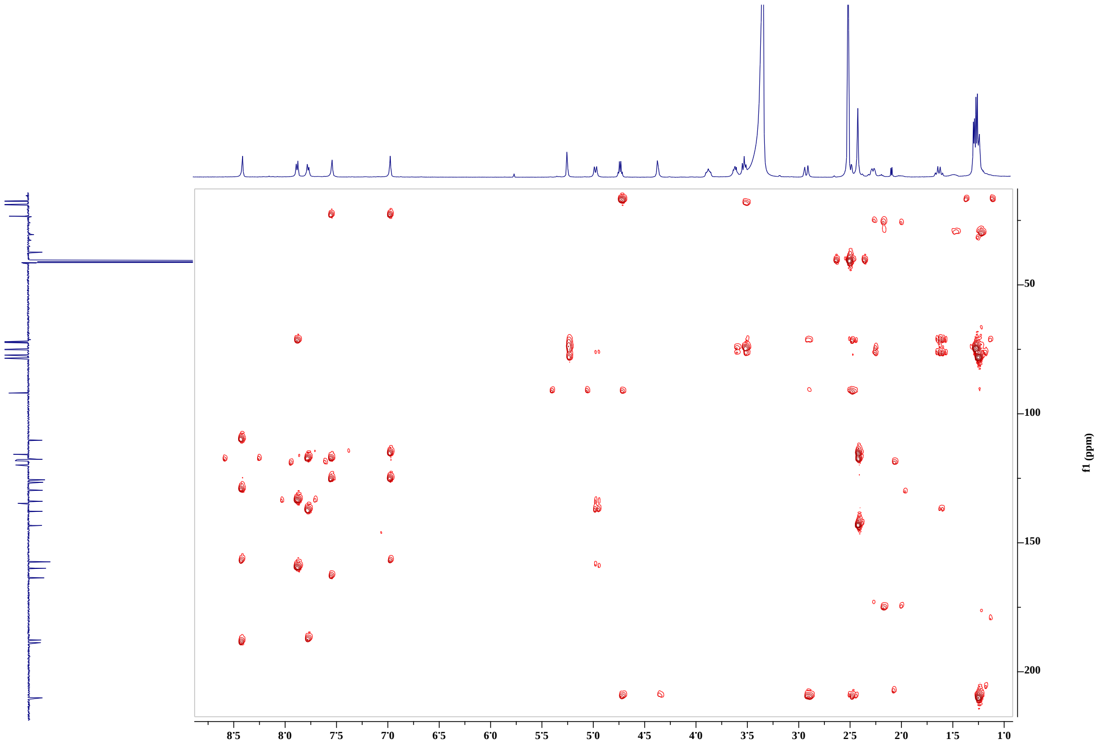

**Figure S21**. $^{1}H$-$^{1}H$ COSY spectrum (500 MHz, DMSO-$d_6$) of **2**.

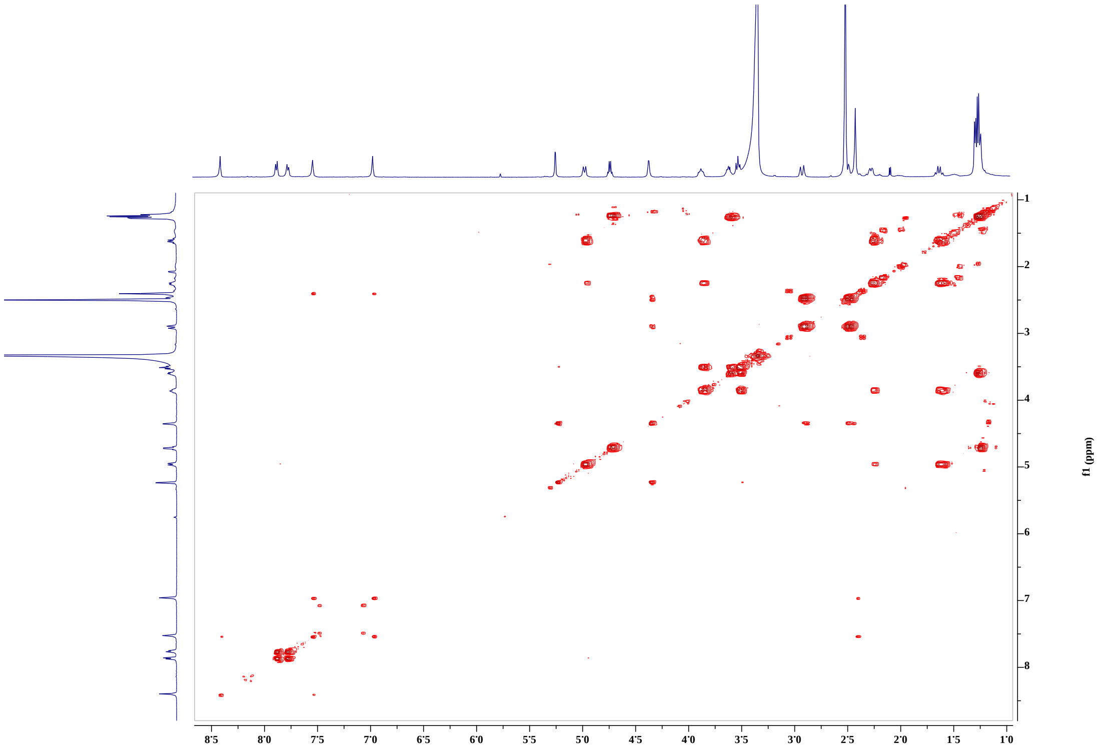


**Figure S22**. NOESY spectrum (500 MHz, DMSO-$d_6$) of **2**.

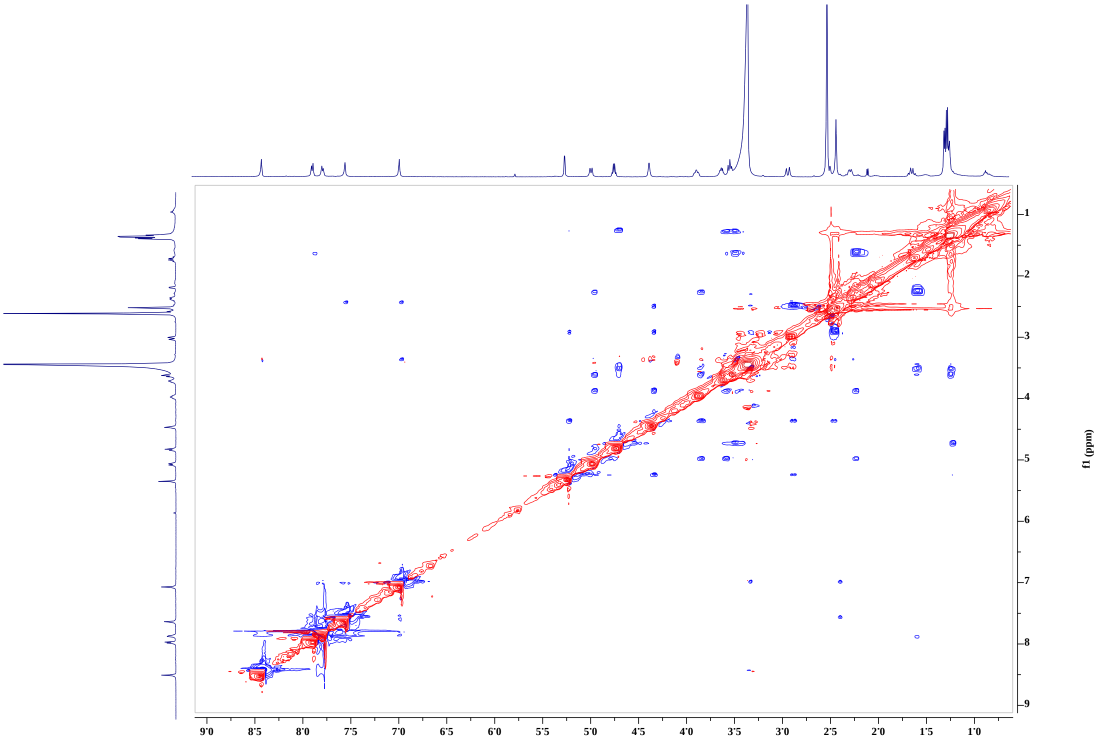

**Figure S23**. HR-ESI-MS of **3**.

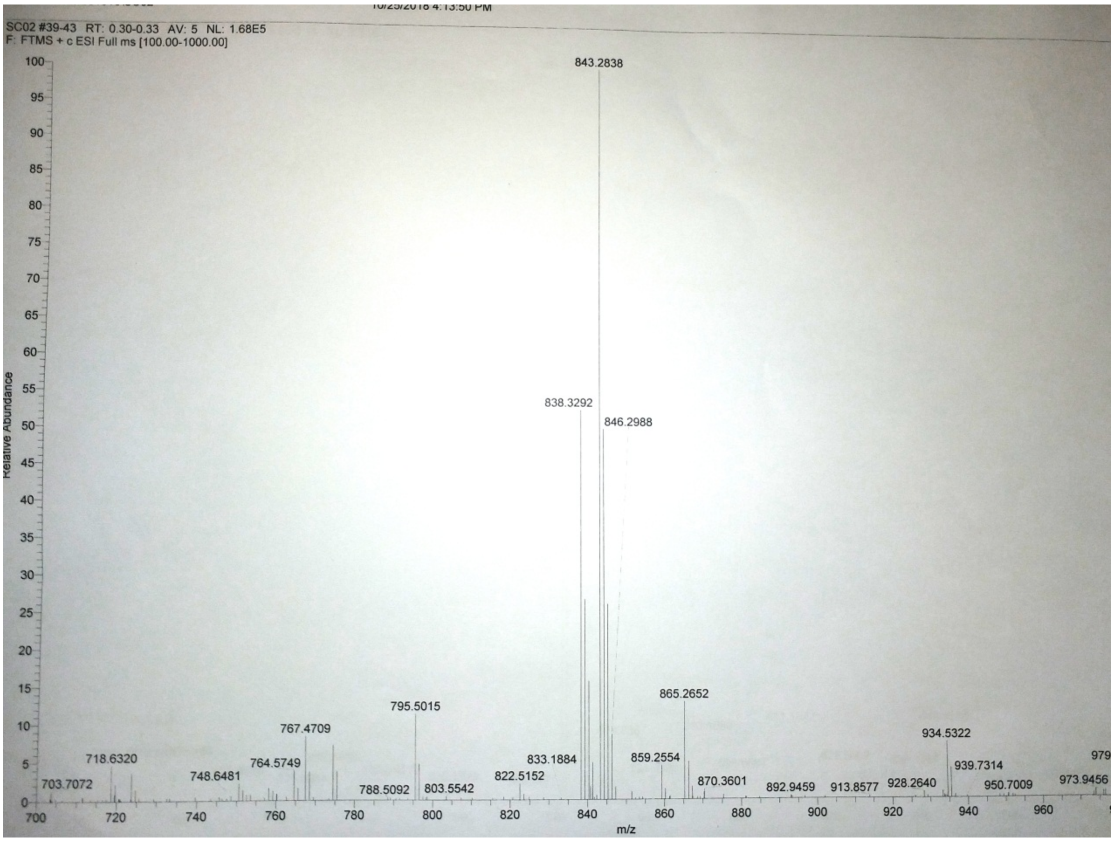


**Figure S24**. $^{1}$H-NMR spectrum (500 MHz, acetone-$d_6$) of **3**.

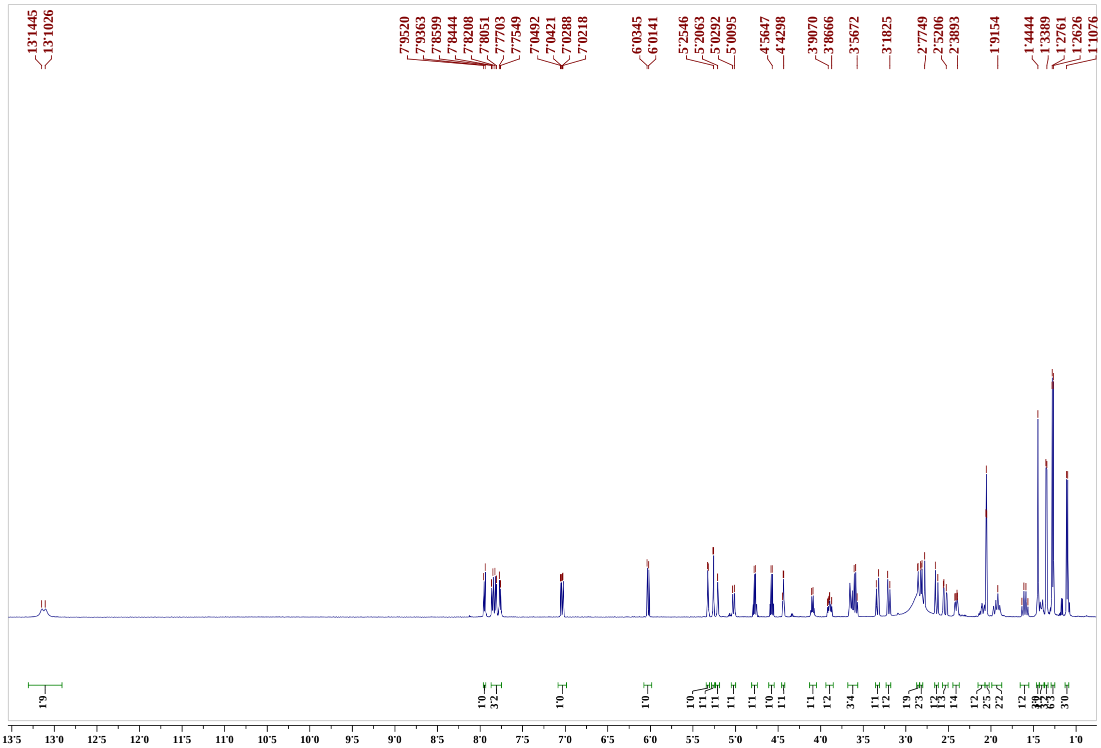

**Figure S25**. $^{13}$C-NMR (APT) spectrum (125 MHz, acetone-d$_6$) of **3**.

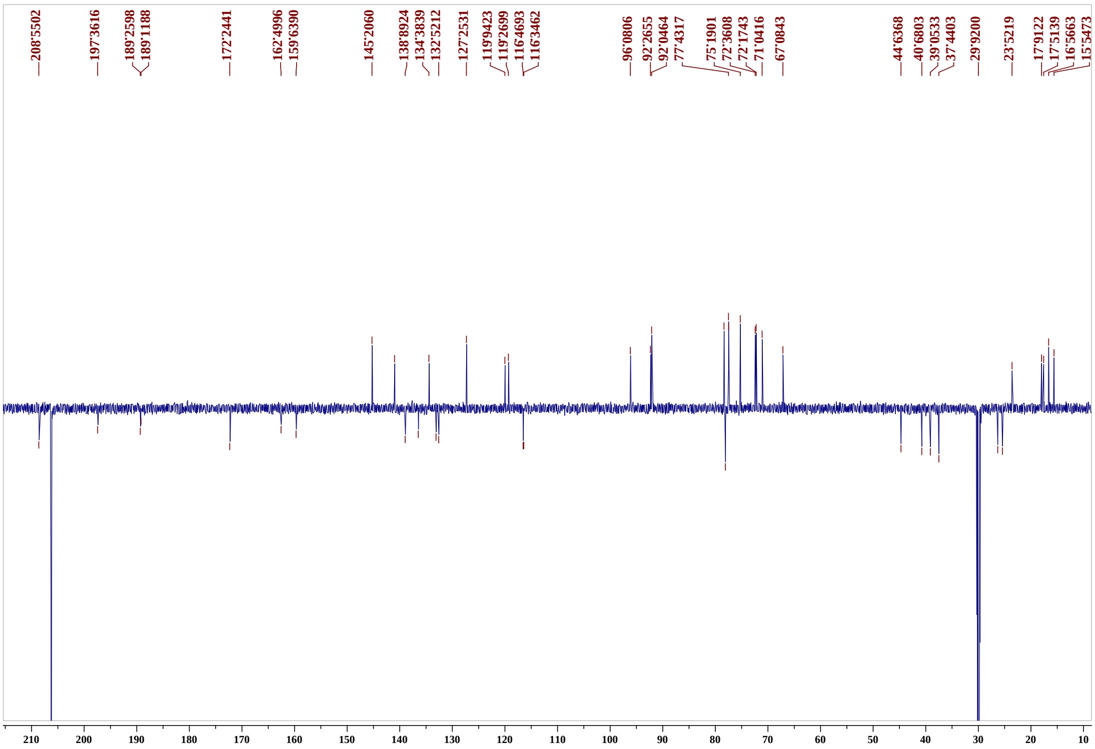


**Figure S26**. HMQC spectrum (500 MHz, acetone-d$_6$) of **3**.

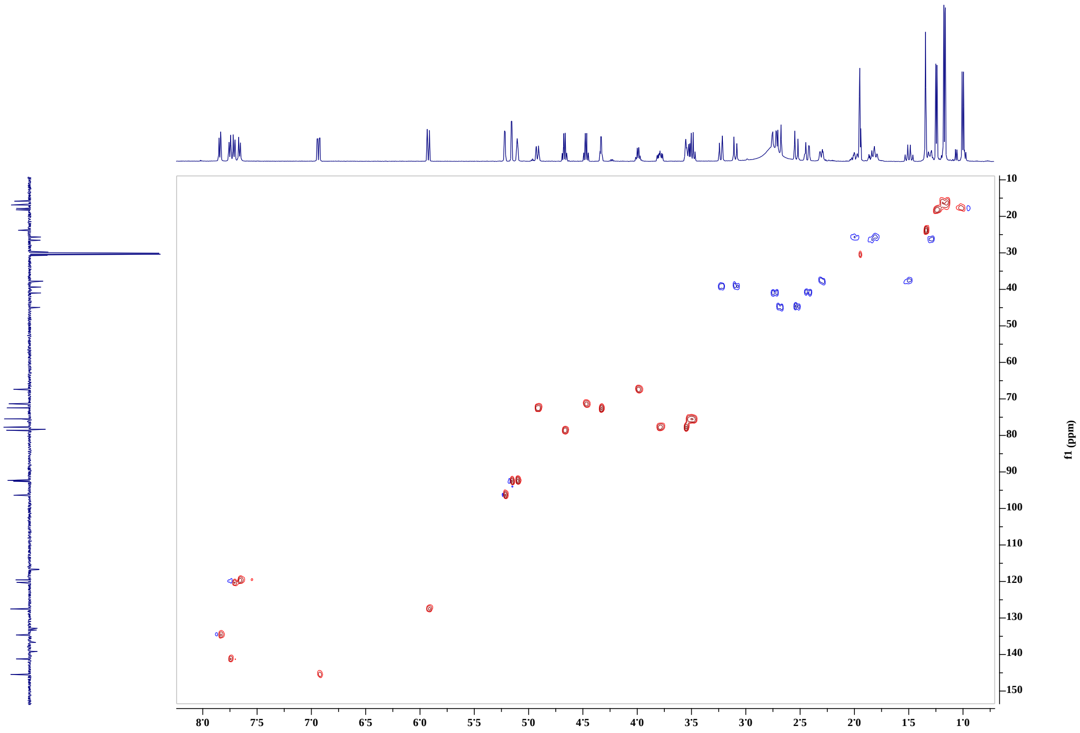

**Figure S27**. HMBC spectrum (500 MHz, acetone-$d_6$) of **3**.

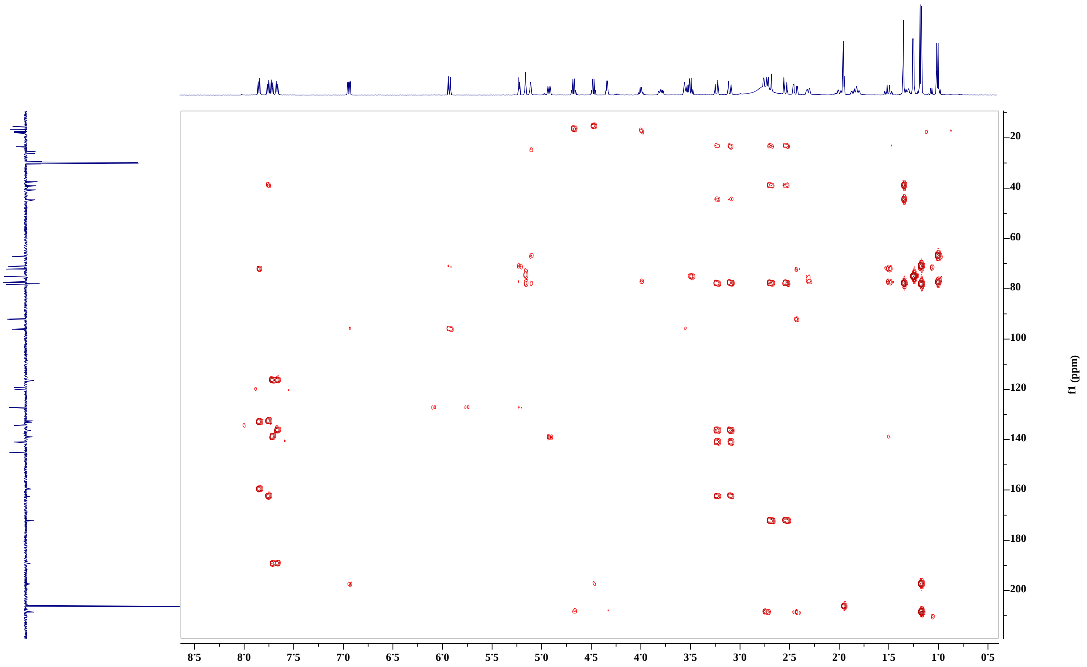


**Figure S28**. $^1$H-$^1$H COSY spectrum (500 MHz, acetone-$d_6$) of **3**.

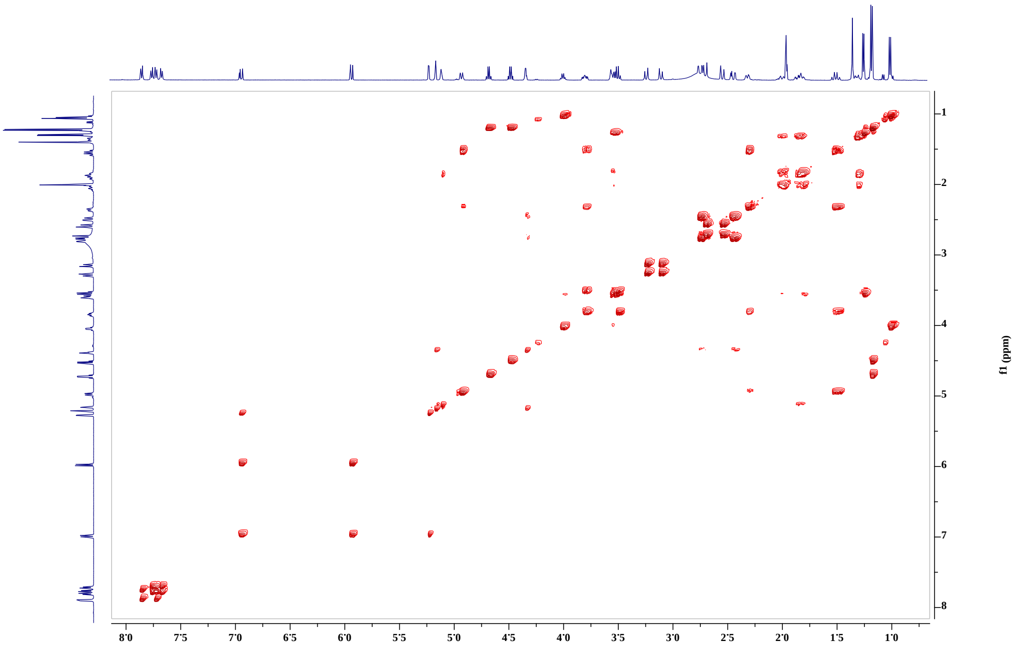

**Figure S29**. NOESY spectrum (500 MHz, acetone-$d_6$) of **3**.

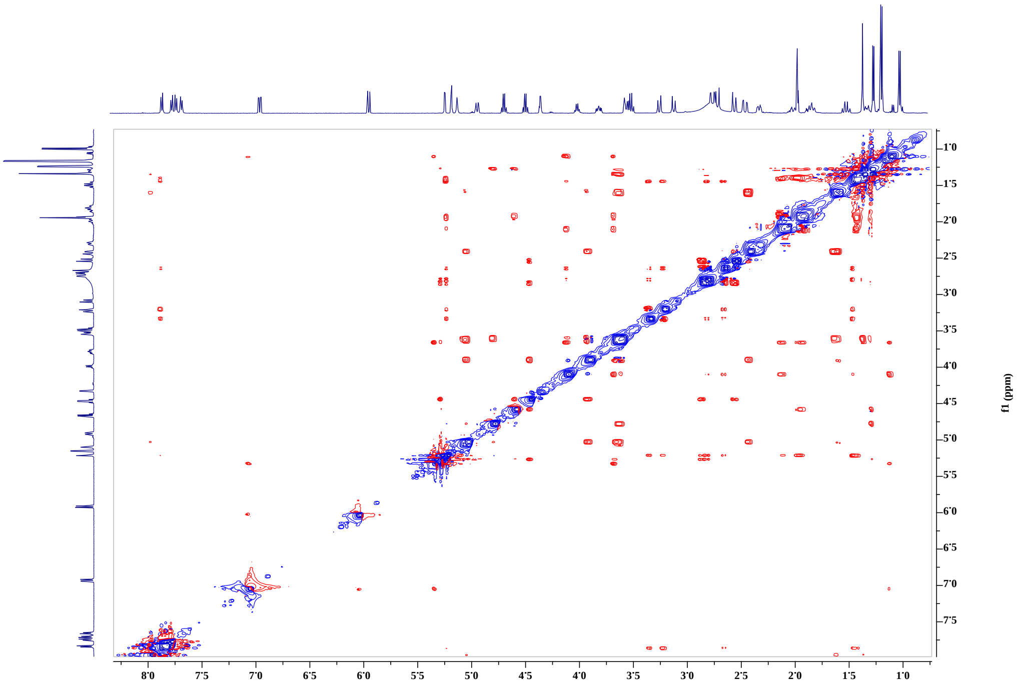

Supplement: Supplementary file 1 [file marinedrugs-16-00470-s001.pdf]
